# The External Globus Pallidus as the Hub of the Auditory Cortico-Basal Ganglia Loop

**DOI:** 10.1523/ENEURO.0161-24.2024

**Published:** 2024-11-26

**Authors:** Ryohei Tomioka, Naoki Shigematsu, Toshio Miyashita, Yukie Takahashi, Mariko Yamamoto, Yumiko Yoshimura, Kenta Kobayashi, Yuchio Yanagawa, Nobuaki Tamamaki, Takaichi Fukuda, Wen-Jie Song

**Affiliations:** ^1^Department of Sensory and Cognitive Physiology, Faculty of Life Sciences, Kumamoto University, Kumamoto 860-8556, Japan; ^2^Morphological Neural Science, Graduate School of Life Sciences, Kumamoto University, Kumamoto 860-8556, Japan; ^3^Department of Anatomy and Neurobiology, Faculty of Life Sciences, Kumamoto University, Kumamoto 860-8556, Japan; ^4^Department of Anatomy, Teikyo University School of Medicine, Tokyo 173-8605, Japan; ^5^Division of Visual Information Processing, National Institute for Physiological Sciences, Okazaki, Aichi 444-8585, Japan; ^6^Section of Viral Vector Development, National Institute for Physiological Sciences, Okazaki 444-8585, Japan; ^7^Department of Genetic and Behavioral Neuroscience, Gunma University Graduate School of Medicine, Maebashi 371-8511, Japan; ^8^Center for Metabolic Regulation of Healthy Aging, Faculty of Life Sciences, Kumamoto University, Kumamoto 860-8556, Japan.

**Keywords:** auditory system, cortico-basal ganglia loop, globus pallidus, medial geniculate body, mouse

## Abstract

The cortico-basal ganglia loop has traditionally been conceptualized as consisting of three distinct information networks: motor, limbic, and associative. However, this three-loop concept is insufficient to comprehensively explain the diverse functions of the cortico-basal ganglia system, as emerging evidence suggests its involvement in sensory processing, including the auditory systems. In the present study, we demonstrate the auditory cortico-basal ganglia loop by using transgenic mice and viral-assisted labelings. The caudal part of the external globus pallidus (GPe) emerged as a major output nucleus of the auditory cortico-basal ganglia loop with the cortico-striato-pallidal projections as its input pathway and pallido-cortical and pallido–thalamo–cortical projections as its output pathway. GABAergic neurons in the caudal GPe dominantly innervated the nonlemniscal auditory pathway. They also projected to various regions, including the substantia nigra pars lateralis, cuneiform nucleus, and periaqueductal gray. Considering the functions associated with these GPe-projecting regions, auditory cortico-basal ganglia circuits may play a pivotal role in eliciting defensive behaviors against acoustic stimuli.

## Significance Statement

The cortico-basal ganglia loops have been extensively studied. However, the auditory cortico-basal ganglia loop has remained elusive. In the present study, we demonstrated a cortico-basal ganglia loop in the auditory system. Our findings highlight GABAergic in the caudal external globus pallidus (GPe) as a major output nucleus in the auditory basal ganglia circuit. We identified principal targets of the caudal GPe, including the nonlemniscal auditory pathway, substantia nigra pars lateralis, cuneiform nucleus, and periaqueductal gray. Our findings provide novel insights into the auditory system and auditory-triggered behavior.

## Introduction

The cortico-basal ganglia loop is known as a fundamental loop circuit in the forebrain. In the classical model of the basal ganglia, the striatum serves as the input nucleus, and the internal globus pallidus (GPi) and substantia nigra pars reticulata (SNR) function as the output nuclei via the various interconnected nuclei (Gerfen and Bolam, 2016). However, recent studies have revealed neuronal diversity within basal ganglia nuclei (Bolam et al., 2000; Mallet et al., 2012; Mastro et al., 2014; Abdi et al., 2015; Hernández et al., 2015; Oh et al., 2017; Abecassis et al., 2020; Ogata et al., 2022; Guilhemsang and Mallet, 2024). It has become evident that GABAergic neurons in the rostral external globus pallidus (GPe) represent a heterogeneous population, with distinct subgroups defined by their gene expression profiles and projection targets (Bolam et al., 2000; Mallet et al., 2012; Abdi et al., 2015; Oh et al., 2017; Abecassis et al., 2020). Notably, some GPe neurons project to the thalamus and cortex (Mastro et al., 2014; Saunders et al., 2015; Abecassis et al., 2020), suggesting that the GPe may serve as an output nucleus within the cortico-basal ganglia loop. This diversity in anatomical and neurochemical profiles suggests a more complex neural circuit than assumed in classical models.

Alexander et al. (1986) proposed five cortico-basal ganglia loops: motor, oculomotor, dorsolateral prefrontal, lateral orbitofrontal, and anterior cingulate gyrus. This model was later developed and conceptualized to process three types of information: motor, limbic, and associative (Joel and Weiner, 1994; Kompoliti and Metman, 2010; Simonyan, 2019). In this framework, the motor and oculomotor loops in Alexander’s model are categorized as motor, the dorsolateral prefrontal and lateral orbitofrontal loops as associative, and the anterior cingulate loop as limbic. In the context of cortico-basal ganglia loops for sensory inputs, Alexander et al. (1986) documented somatosensory inputs within the framework of the motor loop, while Middleton and Strick (1996) demonstrated a cortico-basal ganglia loop associated with higher-order visual areas in primates, involving the cortico–striato–nigro–thalamo–cortical loop circuit, using herpes simplex virus Type 1. Despite these findings, descriptions of cortico-basal ganglia loops for sensory modalities remain limited, with no detailed descriptions related to auditory processing. Interestingly, some researchers have proposed roles of the basal ganglia in auditory and visual processing (Seger, 2013; Lim et al., 2014). In addition, recent systematic anatomical analyses have revealed that almost all cortical areas (including the auditory cortex) send excitatory projections to the striatum (Hintiryan et al., 2016; Hunnicutt et al., 2016; Miyamoto et al., 2018; Foster et al., 2021), suggesting that the cortico-basal ganglia loop is involved in a more diverse range of brain functions than previously concepts for motor, limbic, and associative processes.

Several lines of evidence suggest the presence of a cortico-basal ganglia loop associated with the auditory system. First, in both rodents and humans, the auditory cortex projects to the extreme caudal region of the striatum, known as the tail of the striatum (TS; Oh et al., 2014; Hintiryan et al., 2016; Hunnicutt et al., 2016; Miyamoto et al., 2018; Valjent and Gangarossa, 2021; Sitek et al., 2023). Additionally, recent systematic investigations have revealed that neurons in the TS project to the caudal GPe (Foster et al., 2021; Valjent and Gangarossa, 2021). These anatomical findings suggest that the caudal striato-pallidal pathway constitutes a part of the auditory cortico-basal ganglia loop. Second, recent physiological studies have demonstrated the roles of the corticostriatal pathway in the auditory cortex (Znamenskiy and Zador, 2013; Zhong et al., 2014; Xiong et al., 2015; Ponvert and Jaramillo, 2019; Li et al., 2021). However, the presence of the auditory cortico-basal ganglia loop remains elusive.

In this study, we utilized a variety of anatomical techniques to delineate the cortico-basal ganglia loop in the auditory system. Our findings not only reveal the neuronal circuits in this loop but also elucidate its connections to other brain regions. This intricate connectivity provides insight into how auditory information is processed in the brain. Overall, our study contributes to the anatomical understanding of the cortico-basal ganglia loop in the auditory system.

## Materials and Methods

All surgical procedures were performed in accordance with the National Institutes of Health (NIH) “Guidelines for the Care and Use of Laboratory Animals” (NIH publication 86–23), and all protocols were approved by the Kumamoto University Animal Experiment Committee and the Experimental Animal Committee of the National Institute for Physiological Sciences. All efforts were made to minimize animal suffering and to reduce the number of animals used.

The animals (23–30 g at the time of injection) were housed in a ventilated room under standard conditions, including an air-conditioned environment at 20–25°C and 40–60% humidity, with a 12 h light/dark cycle. Adult male C57BL/6J mice (*n* = 9; SLC, Hamamatsu), glutamate decarboxylase 67 (GAD67)-Cre knock-in mice (*n* = 16; bred in the animal facility of Kumamoto University; Higo et al., 2009; Tomioka et al., 2015), GAD67-green fluorescent protein (GFP) knock-in mice (*n* = 14; bred in the animal facility of Kumamoto University; Tamamaki et al., 2003), and vesicular GABA transporter (VGAT)-Cre knock-in mice (RRID: IMSR_JAX:028862) that were back-crossed with the C57BL/6J strain (*n* = 12; bred in the animal facility of National Institute for Physiological Sciences) were used in this study.

### Production of EnvA pseudotyped rabies virus expressing mCherry

The pseudotyped rabies virus, EnvA-HEP-ΔG-mCherry, was used to label presynaptic neurons. EnvA-HEP-ΔG-mCherry was produced as previously described protocols (Osakada et al., 2011; Mori and Morimoto, 2014). Briefly, to produce glycoprotein-deleted rabies virus HEP strain (HEP-ΔG-mCherry), the plasmid containing mCherry and the entire rabies virus genome without rabies glycoprotein and each plasmid containing rabies nucleoprotein, phosphoprotein, and polymerase genes were transfected to BHT-T7/9 cells (TransIT-LT1; Takara Bio). The cell culture plates were incubated for 2–4 d at 32°C in an atmosphere containing 5% CO_2_. Once the number of mCherry-positive cells increased, the culture supernatant was collected and transferred to BHK-SiG cells, which express SADcvsG glycoproteins, to raise the virus titer of HEP-ΔG-mCherry virus. The cell culture medium containing HEP-ΔG-mCherry virus was collected and transferred to BHK-Lv-EiG cells, which express avian sarcoma leucosis virus envelope protein (EnvA), to produce pseudotyped rabies virus, EnvA-HEP-ΔG-mCherry. The cell culture plates were incubated for 4–5 d at 32°C in an atmosphere containing 3% CO_2_. EnvA-HEP-ΔG-mCherry virus was collected from the cell culture medium by centrifugation. The contamination of unpseudotyped rabies virus HEP-ΔG-mCherry in the concentrated virus was examined using HEK293T. Infection of unpseudotyped rabies virus was not confirmed in vitro. We confirmed an infectious titer of EnvA-HEP-ΔG-mCherry virus in 293-TVA cells.

### Surgical procedures and fixation

Each animal was deeply anesthetized with a mixture of ketamine (80  mg/kg, i.p.; Fujita) and xylazine (8  mg/kg; Elanco) and placed in a stereotaxic frame (Narishige). A craniotomy was performed at the appropriate skull location. All injections were performed by air pressure using a custom-made syringe pump connected to a glass capillary needle (Calibrated micropipettes 2-000-001, Drummond Scientific). For the anterograde transneuronal tracing experiment, we injected 0.2 µl of adeno-associated virus [AAV; AAV1-CMV-HI-eGFP-Cre: 2.8 × 10^12^ viral genomes per µl (vg/ml), Addgene Viral Prep#105545-AAV1; Addgene] into the primary auditory cortex (A1; bregma, −3.0 mm; lateral, 4.5 mm; depth, 0.5 mm) and 0.1 µl of AAV2-CAG-FLEX-tdTomato (1.2 × 10^13^ vg/ml, University of North Carolina Vector Core) into the TS (bregma, −1.3 mm; lateral, 3.6 mm; depth, 2.7 mm). The survival time for the anterograde transneuronal tracing experiment was 3 weeks. To visualize axonal fibers from GPe GABAergic neurons, we injected 0.08 µl of AAV2-CAG-FLEX-GFP (2.2 × 10^13^ vg/ml, University of North Carolina Vector Core) into the GPe of GAD67-Cre mice (bregma, −1.2 mm; lateral, 2.7 mm; depth, 3.2 mm). The survival time for the anterograde experiment was 3 weeks. We injected 0.02 µl of Fluoro-Gold (FG; 4% dissolved in water; Fluorochrome) into GAD67-GFP mice as a retrograde tracer. The FG-injection sites are the temporal association cortex (bregma, −3.0 mm; depth, 0.5 mm from the cortical surface at a 50° angle from the horizontal plane), medial geniculate body (MGB; bregma, −3.0 mm; lateral, 2.0 mm; depth, 3.0 mm), and cuneiform nucleus (CnF; bregma, −5.0 mm; lateral, 1.5 mm; depth, 2.0 mm). The survival time for the retrograde experiments was 3 d. To identify the presynaptic neurons of GPe GABAergic neurons, we injected 0.2 µl of a virus mixture (AAVretro-CA-FLEX-SADcvsG, 2.0 × 10^12^ vg/ml, and AAVretro-EF1a-FLEX-GFP-2A-TVA, 9.8 × 10^11^ vg/ml in National Institute for Physiological Sciences, Section of Viral Vector Development) into the temporal association cortex of VGAT-Cre mice (bregma, −3.0 mm; depth, 0.5 mm from the cortical surface at a 50° angle from the horizontal plane). Three weeks later, 0.2 µl of pseudotyped rabies virus (EnvA-HEP-ΔG-mCherry, 2.3 × 10^8^ infectious units/ml) was injected into the caudal GPe (bregma, −1.2 mm; lateral, 2.7 mm; depth, 3.2 mm). The survival time after the second injection was 7 d. We performed two control experiments using pseudotyped rabies virus. To confirm the contamination of unpseudotyped rabies virus in vivo, AAVretro-CA-FLEX-SADcvsG and AAVretro-EF1a-FLEX-GFP-2A-TVA were injected into A1 of C57BL/6J mice, following the injection of pseudotyped rabies virus into the same area. In another control experiment, to confirm the distribution of starter cells, AAVretro-EF1a-FLEX-GFP-2A-TVA were injected into the temporal association cortex of VGAT-Cre mice, following the injection of pseudotyped rabies virus into the caudal GPe.

For confocal microscopic examination, mice were perfused with 0.05 M phosphate-buffered saline [PBS; 0.9% (*w*/*v*) saline buffered with 0.05 M sodium phosphate], pH 7.4, followed by 4% formaldehyde in PBS under deep anesthesia (ketamine, 120 mg/kg; xylazine, 12 mg/kg). The brains were cryoprotected with 30% sucrose in PBS, and 50-μm-thick coronal sections were cut using a cryostat (CM1950, Leica Biosystems). For electron microscopic examination, mice were deeply anesthetized and then perfused with PBS, pH 7.4, followed by 50 ml of a mixture of 0.1% glutaraldehyde and 4% paraformaldehyde in 0.1 M PB at room temperature. Brains were removed from the skull and stored overnight in 4% paraformaldehyde in 0.1 M PB at 4°C. The fixative was replaced by PBS. Serial 40-µm-thick coronal sections were cut using a vibrating microtome (model TTK-3000, Dosaka). Histological analysis was conducted according to the mouse brain atlas to identify specific brain structures (Franklin and Paxinos, 2007).

### Fluorescent immunostaining

To identify the anatomical or neurochemical properties, we performed immunostaining using the following primary antibodies (see the detailed information in Table 1): a mouse anti-calretinin (CR) antibody (Swant), goat anti-choline acetyltransferase (ChAT) antibody (Merck), rabbit anti-FG antibody (Merck), goat anti-forkhead box protein P2 (FoxP2) antibody (Santa Cruz Biotechnology), rabbit anti-GFP antibody (Tomioka et al., 2015), mouse anti-LIM Homeobox 6 (Lhx6) antibody (Santa Cruz Biotechnology), rabbit anti-NeuN antibody (Abcam), guinea pig anti-parvalbumin (PV) antibody (NITTOBO MEDICAL), mouse anti-PV antibody (Swant), mouse anti-protein kinase C δ (PKCδ) antibody (BD Transduction Laboratories), rabbit anti-prodynorphin (PD) antibody (Abcam), rabbit anti-preproenkephalin (PPE) antibody (Neuromics), rabbit anti-synaptophysin antibody (NITTOBO MEDICAL), and mouse anti-tyrosine hydroxylase (TH) antibody (Santa Cruz Biotechnology). After blocking with PBS containing 0.3% Triton X-100 and 1% normal donkey serum for 1 h at room temperature, sections from at least three animals were incubated with primary antibodies overnight at room temperature in a blocking solution. The sections were incubated with an appropriate set of secondary antibodies labeled with fluorescent dyes at different wavelengths for 2 h at room temperature (Table 2). Images were obtained using a confocal laser scanning microscope (FV3000; Olympus) with appropriate filters for Alexa Fluor 488 (laser wavelength, 488 nm; detection wavelength, 500–540  nm), Alexa Fluor 555 (laser wavelength, 561 nm; detection wavelength, 570–620 nm), or Alexa Fluor 647 (laser wavelength, 640 nm; detection wavelength, 650–750 nm). Images were merged using Canvas 12 (Canvas GFX), and some were displayed in a pseudocolor format.

**Table 1.** List of primary antibodies used in this study

Primary antibody	Dilution	Host species
CR (Swant, 6B3)	1:1,000	Mouse
CR (Swant, 7699/4)	1:5,000	Rabbit
ChAT (Merck, AB144P)	1:1,000	Goat
FG (Merck, AB153-I)	1:2,000	Rabbit
FoxP2 (Santa Cruz Biotechnology, sc-21069)	1:1,000	Goat
GFP (Nacalai, GF200)	1:1,000	Rat
GFP (Tomioka et al.,2015)	1 µg/ml	Rabbit
Lhx6 (Santa Cruz Biotechnology, sc-271433)	1:100	Mouse
NeuN (Abcam, ab177487)	1:1,000	Rabbit
PV (NITTOBO MEDICAL, MSFR105250)	1:200	Guinea pig
PV (Swant, 235)	1:500	Mouse
PKCδ (BD Transduction Laboratories, 610398)	1:500	Mouse
PD (Abcam, ab11137)	1:100	Rabbit
PPE (Neuromics, RA14124)	1:8,000	Rabbit
Synaptophysin (NITTOBO MEDICAL, MSFR105660)	1:200	Rabbit
Tyrosine hydroxylase (Santa Cruz Biotechnology, sc-25269)	1:500	Mouse

**Table 2.** List of secondary antibodies used in this study

Second antibody	Dilution source		Catalog#
Alexa Fluor 488-conjugated donkey anti-mouse IgG	1:1,000	Thermo Fisher Scientific	A-32766
Alexa Fluor 488-conjugated donkey anti-rabbit IgG	1:1,000	Thermo Fisher Scientific	A-32790
Alexa Fluor 555-conjugated donkey anti-mouse IgG	1:1,000	Thermo Fisher Scientific	A-31570
Alexa Fluor 555-conjugated streptavidin	1:1,000	Thermo Fisher Scientific	S-21381
Alexa Fluor 647-conjugated donkey anti-mouse IgG	1:1,000	Thermo Fisher Scientific	A-32787
Alexa Fluor 647-conjugated donkey anti-guinea pig IgG	1:1,000	Thermo Fisher Scientific	A-21450
Alexa Fluor 647-conjugated donkey anti-rabbit IgG	1:1,000	Thermo Fisher Scientific	A-32795
Alexa Fluor 647-conjugated streptavidin	1:500	Jackson ImmunoResearch Laboratories	016–600-084
Biotiny-SP-conjugated donkey anti-rat IgG	1:250	Jackson ImmunoResearch Laboratories	712-065-153
Biotiny-SP-conjugated donkey anti-goat IgG	1:1,000	Thermo Fisher Scientific	A-16009
Biotiny-SP-conjugated goat anti-guinea pig IgG	1:1,000	Thermo Fisher Scientific	A-18779
Peroxidase anti-peroxidase	1:500	Jackson ImmunoResearch Laboratories	323-005-024
Rhodamine red-conjugated donkey anti-rabbit IgG	1:200	Jackson ImmunoResearch Laboratories	711-295-152

### Correlated confocal laser scanning light microscopy (CLSM)–electron microscopy (EM)

Five mice were used for CLSM–EM examination. The CLSM–EM method was performed as described previously with slight modifications (Fukuda, 2017; Shigematsu et al., 2019; Ogata et al., 2022). After cryoprotection with 25% sucrose in PBS, the sections placed on aluminum foil were quickly frozen in liquid nitrogen vapor and rapidly thawed with 25% sucrose in PBS. The sections were incubated with 1% bovine serum albumin (Merck) and 0.1% sodium azide in PBS for 1 h, followed by incubation with a mixture of primary antibodies containing rabbit anti-CR (1:5,000; Swant) antibody and rat anti-GFP (1:500; Nacalai) at 4°C for 4 d. After rinsing several times in PBS, the sections were incubated with biotinylated anti-rat IgG (1:250; Jackson ImmunoResearch Laboratories) at 4°C overnight, followed by incubation with Alexa Fluor 647-conjugated streptavidin (Molecular Probes) and Rhodamine Red-conjugated anti-rabbit IgG (1:200; Jackson ImmunoResearch Laboratories) at 4°C overnight.

Appositions of GFP-positive boutons on CR-positive neurons were examined using a 40× objective lens. After CLSM, coverslips were removed, and the sections were treated with the ABC method (Vector Laboratories) using the DAB-Ni peroxidase reaction to visualize GFP-positive axonal fibers. After the reaction, the sections were rinsed overnight in PBS containing 0.1% sodium azide at 4°C. After several rinses in PBS, the sections were incubated with rabbit peroxidase anti-peroxidase complex (Jackson ImmunoResearch Laboratories, 1:500) in PBS at 4°C overnight. After several rinses in PBS, sections were treated with a DAB-peroxidase reaction, followed by OsO_4_ postfixation, en bloc staining with 1.5% uranyl acetate, dehydration in ethanol, and embedding in Epon-Araldite. Synaptic connections between GFP-positive boutons and CR-positive neurons at the apposition sites observed by CLSM were examined by EM (HT7700, Hitachi).

### Image data analysis

Five mice were used for the quantitative measurements of axonal fibers. Image analysis was performed using a previously described method, with slight modifications (Tomioka et al., 2023). Similar coronal sections were analyzed for the quantitative measurement of GFP-labeled axonal fibers from the GPe. Images were obtained using a confocal laser scanning microscope (FV3000) with a 10× objective lens at a 2,048 × 2,048 pixel resolution. All images were analyzed using the MATLAB software (R2019b; MathWorks). Briefly, the boundaries of the MGB subdivisions were defined according to PKCδ immunoreactivity (Tomioka et al., 2023). The area of each subdivision was calculated. The raw images of GFP-positive signals were converted into binary images using one threshold per section. The summed area of GFP-positive signals in each subdivision was calculated. Finally, the occupancy of GFP-positive signals in each MGB subdivision was computed. Friedman’s test and Bonferroni’s post hoc test were used for multiple comparisons.

Six mice were used for quantitative measurement of Lhx6 immunoreactivity. Images were obtained using a confocal laser scanning microscope (FV3000) with a 40× objective lens at a 2,048 × 2,048 pixel resolution. We measured the intensity of Lhx6 immunoreactivity in the caudal GPe. We calculated the average intensities of Lhx6 immunoreactivity in PV-positive and PV-negative neurons in each animal. A two-tailed paired *t* test was used for statistical analysis. Statistical significance was set at *p* < 0.05.

## Results

### Auditory striatal projecting neurons

**Figure 1.**
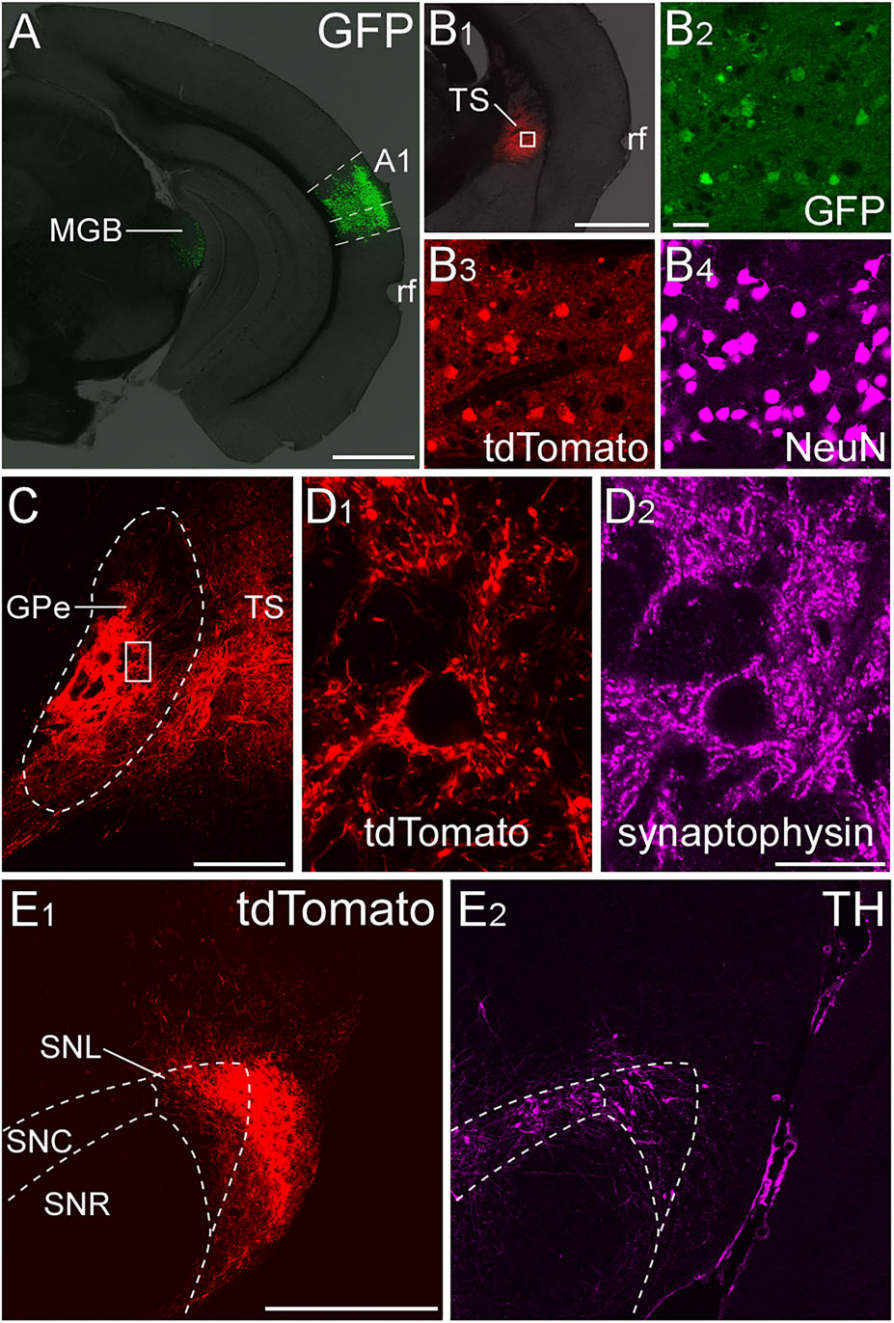
Axonal fibers of striatal neurons receiving auditory corticostriatal projections. ***A***, AAV1-CMV-HI-eGFP-Cre was injected into A1. The injection site was mainly confined in the A1. The rhinal fissure (rf) is shown as a brain region marker. ***B*_1_**, AAV2-CAG-FLEX-tdTomato was injected into the TS. ***B*_2_*–B*_4_**, The enlarged image of the area marked by the rectangle of ***B*_1_**. The tdTomato-expressing neurons were found within the TS. ***C***, Dense tdTomato-positive striatal fibers were in the caudal GPe. ***D*_1_**, The enlarged image of the area marked by the rectangle of ***C***. The distribution of tdTomato-positive axon terminals was similar to that of synaptophysin immunoreactivity in the caudal GPe (***D*_2_**). ***E*_1_**, tdTomato-positive striatal fibers were in the SNL, but not in the SNc or reticulata (SNR). ***E*_2_**, TH-immunoreactivity was used to define the boundaries of the substantia nigra. See Extended Data Figure 1-1 for more details. Scale bars: 1.0 mm in ***A*** and ***B*_1_**, 50 μm in ***B*_2_**, 250 µm in ***C***, 25 µm in ***D***, and 500 µm in ***E***.

Recent systematic studies utilizing large tracing datasets have provided detailed maps of corticostriatal and striato-pallidal projections, suggesting the existence of a neural network for auditory information processing in the basal ganglia (Hintiryan et al., 2016; Hunnicutt et al., 2016; Foster et al., 2021; Valjent and Gangarossa, 2021; Ogata et al., 2022). First, we examined auditory-related striatal projections using anterograde transneuronal tracing. Because AAV serotype 1 allows for anterograde transneuronal labeling (Zingg et al., 2017), Cre recombinase was introduced into striatal neurons by injecting AAV1-CMV-HI-eGFP-Cre into A1 (Fig. 1*A*). With an additional injection of AAV2-CAG-FLEX-tdTomato into the TS, postsynaptic striatal neurons were labeled with tdTomato in a Cre-dependent manner (Fig. 1*B*). The distribution of tdTomato-labeled axonal fibers was predominantly observed in the caudal GPe (Fig. 1*C,D*), followed by the substantia nigra pars lateralis (SNL; Fig. 1*E*; Extended Data Fig. 1-1), but did not extend to the GPi and SNR (data not shown). Since the GPe would be on the major pathway of the auditory cortico-basal ganglia loops, we extensively analyzed the neural circuit via the caudal GPe (−1.06 to −1.7  mm from the bregma along the rostrocaudal axis) in the present study.

10.1523/ENEURO.0161-24.2024.f1-1 Figure 1-1 Axonal fibers of striatal neurons receiving corticostriatal projections from the primary auditory cortex. Striatal axonal fibers, which were labeled with tdTomato, formed close synaptic appositions (arrows) with TH-positive dopaminergic (A, B) or PV-positive GABAergic neurons (C, D) in the SNL. Scale bar = 500 µm in A and C, 10 µm in B and D. Download Figure 1-1, TIF file.

### Anterograde tracing from GABAergic neurons in the caudal GPe

**Figure 2.**
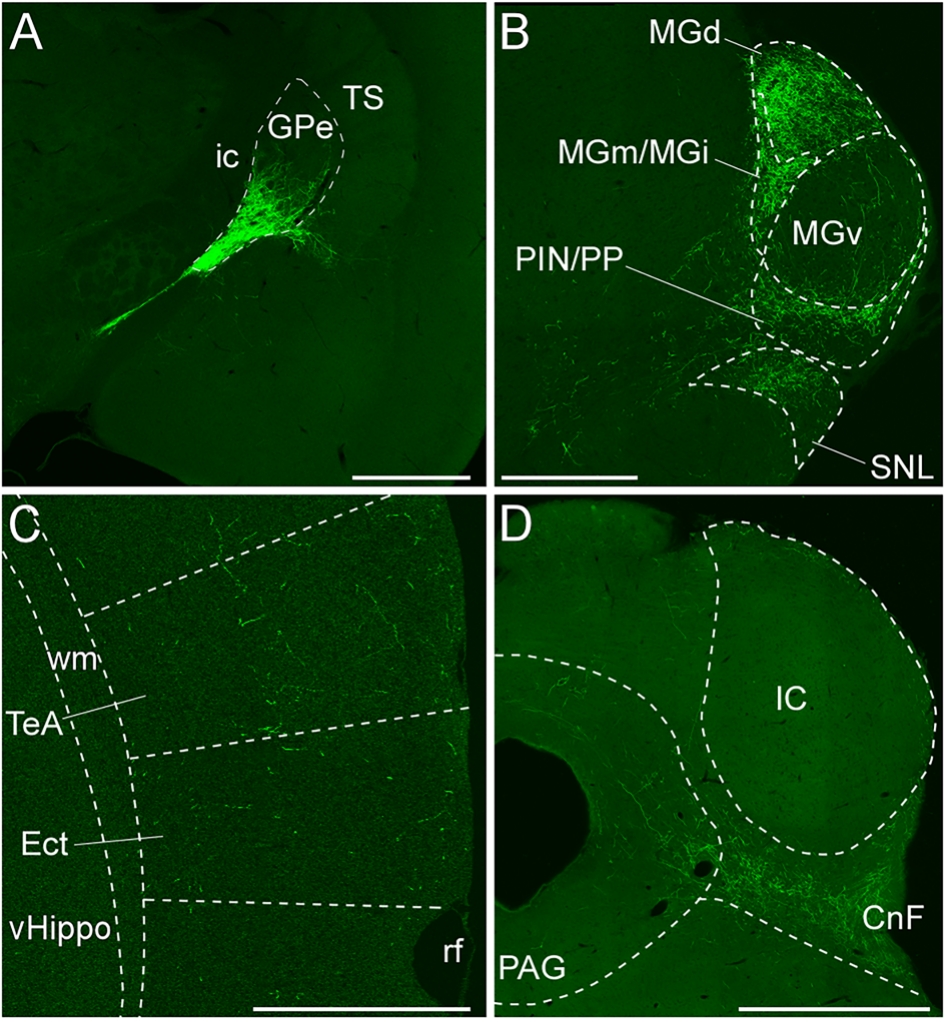
Axonal fibers from GABAergic neurons in the caudal GPe. ***A***, AAV2-CAG-FLEX-GFP was injected into the caudal GPe of the GAD67-Cre mouse. GFP-immunoreactive neurons were confined in the caudal GPe. The internal capsule (ic) is shown as a brain region marker. GFP-immunoreactive axonal fibers were distributed in the several subdivisions of MGB and SNL in ***B***, in the temporal association cortex (TeA) and Ect in ***C***, and CnF and PAG in ***D***. Scale bars: 1.0 mm in ***A*** and ***D*** and 0.5 mm in ***B*** and ***C***.

We demonstrated that the caudal GPe is involved in the auditory cortico-basal ganglia loop. In addition, accumulating neuroanatomical and physiological evidence indicates that the caudal GPe is related to auditory function (Moriizumi and Hattori, 1992; Shammah-Lagnado et al., 1996; Zhong et al., 2014; Chavez and Zaborszky, 2017; Foster et al., 2021). We next examined the axonal projections from GABAergic neurons in the caudal GPe. AAV2-CAG-FLEX-GFP was injected into the caudal GPe of the GAD67-Cre mice. The injection sites were localized within the GPe in five mice, as illustrated and further analyzed (Fig. 2*A*). The GFP-immunoreactive axonal fibers were found mainly in and around the MGB (Fig. 2*B*), moderately in the CnF (Fig. 2*D*), and sparsely in the periaqueductal gray (PAG; Fig. 2*D*), temporal association area (TeA), and ectorhinal cortex (Ect; Fig. 2*C*). Pallido-cortical fibers were widely distributed across the rostral–caudal axis of the TeA/Ect. Our anatomical evidence aligns with the previous study using anterograde and retrograde tracing (Shammah-Lagnado et al., 1996), which demonstrated that GPe GABAergic neurons project to the lateral hypothalamus and substantia nigra pars lateralis. Thus, the present study is the first to demonstrate that GPe GABAergic neurons also project to the CnF and cortical areas.

**Figure 3.**
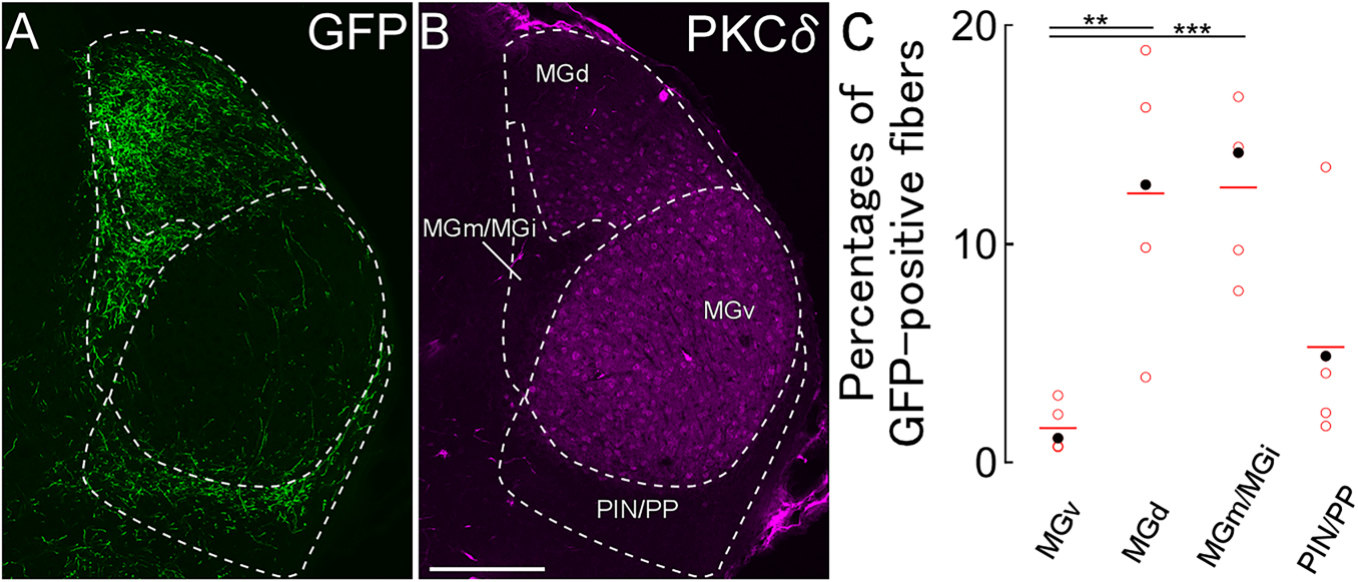
Axonal fibers of GPe GABAergic neurons in the MGB. ***A, B***, GFP-positive fibers were mainly distributed in MGd, MGm/MGi, and PIN/PP. The boundaries for each subdivision of MGB were defined by PKCδ immunoreactivity. ***C***, The densities of GFP-immunoreactive fibers in each subdivision. Each dot indicates the percentage of each mouse. Filled dots are derived from ***A***. MGd and MGm/MGi densities were higher than those of MGv (**p < 0.01; ***p < 0.005). Scale bar, 250 µm.

We further analyzed the densities of GFP-immunoreactive axonal fibers in four subdivisions of the MGB according to anatomical and neurochemical properties (Llano and Sherman, 2008; Márquez-Legorreta et al., 2016; Tomioka et al., 2023). These subdivisions include the ventral division of the MGB (MGv), dorsal division of the MGB (MGd), medial and internal division of the MGB (MGm/MGi; Tomioka et al., 2023), and posterior intralaminar nucleus and peripeduncular nucleus (PIN/PP). The PKCδ immunoreactivity exhibited distinct boundaries of MGB subnuclei, with uniform PKCδ immunoreactivity in MGv, gradient PKCδ immunoreactivity in MGd, and poor PKCδ immunoreactivity in MGm/MGi and PIN/PP (Fig. 3*B*). The distribution of GFP-immunoreactive axonal fibers was primarily in the MGd and MGm/MGi, secondary in the PIN/PP, and rarely in the MGv (Fig. 3*A*,*C*). Considering that the MGv is on the lemniscal pathway of the central auditory system while the others are on nonlemniscal pathways, our findings suggest that GABAergic neurons in the caudal GPe exert an inhibitory effect on the nonlemniscal auditory pathway.

**Figure 4.**
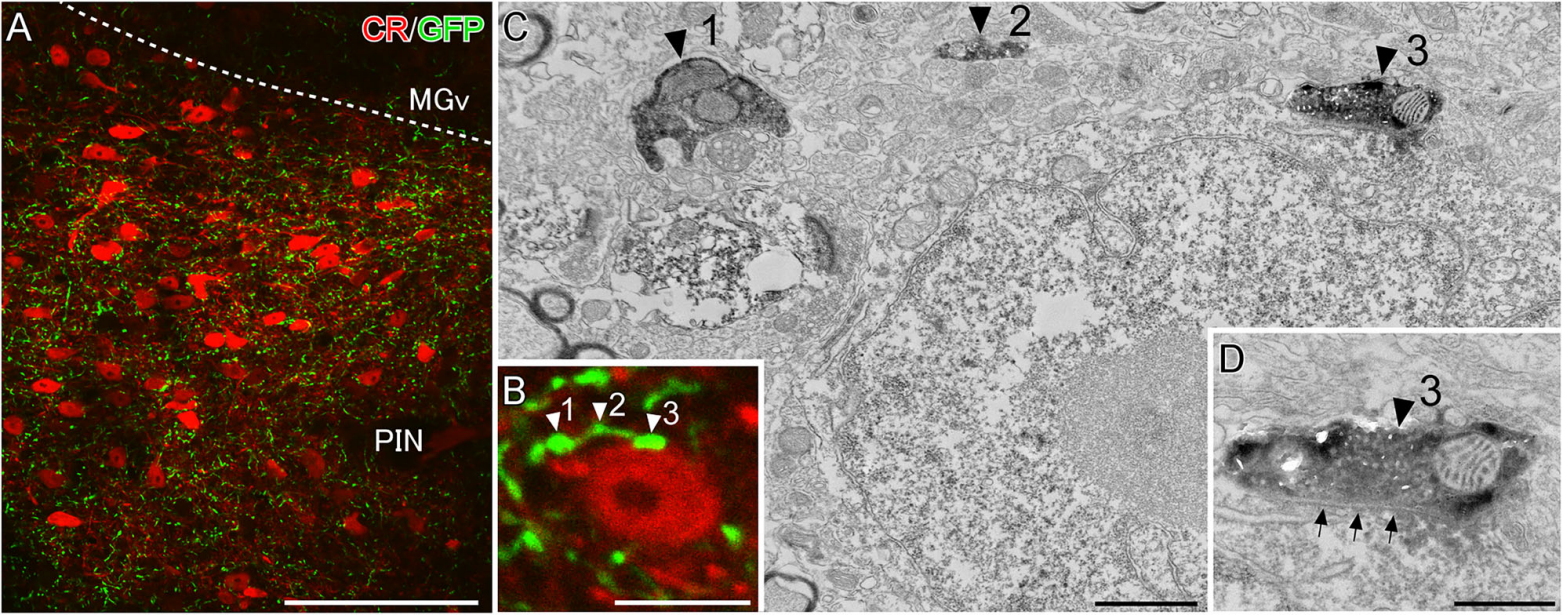
GPe GABAergic fibers forming synaptic contacts on CR-expressing neurons in the PIN. ***A***, CR-immunoreactive neurons and numerous GFP-expressing GPe GABAergic fibers are accumulated in the PIN. ***B***, Higher-magnification image of a CR-immunoreactive neuron analyzed by CLSM–EM. ***C***, An EM image of the same structure shown in ***B***. GFP and CR immunoreactivities are visualized using DAB-nickel and DAB, respectively. All three axonal boutons (black arrowheads 1–3) correspond to boutons in ***B*** (white arrowheads 1–3). ***D***, Arrows indicate that a GFP-immunoreactive axon terminal formed a symmetrical synaptic contact on the CR-immunoreactive soma. Scale bars: 100 µm in ***A***, 10 µm in ***B***, 1  µm in ***C***, and 0.5 µm. See Extended Data Figures 4-1 and 4-2 for more details.

The distribution pattern of GFP-immunoreactive axonal fibers in and around the MGB was similar to that of CR-expressing neurons (Llano and Sherman, 2008; Márquez-Legorreta et al., 2016; Tomioka et al., 2023). Of note, several studies have reported CR-expressing neurons in both the MGd and PIN/PP project to the temporal cortices including TeA/Ect and ventral part of the secondary auditory cortex (AuV) and lateral amygdala (Cai et al., 2019; Barsy et al., 2020; Belén Pardi et al., 2020). We next examined whether GABAergic neurons in the caudal GPe form synaptic contacts with CR-immunoreactive neurons. The CR-immunoreactive neurons were mainly distributed in the MGd and PIN/PP (Fig. 4-1), which is consistent with previous reports (Lu et al., 2009). Under CLSM observation, GFP-immunoreactive puncta of GPe GABAergic projection neurons were found in close apposition with the CR-immunoreactive somata in the PIN/PP and MGd (Fig. 4; Extended Data Fig. 4-2, respectively). To assess potential synaptic connections by CLSM, we performed double-labeled immunoelectron microscopy. CR and GFP immunoreactivities were visualized DAB and DAB-nickel as chromogenic agents, respectively (Fig. 4). The GFP-positive axon terminals forming symmetrical synapses on the CR-immunoreactive soma were observed in the PIN/PP (Fig. 4) and MGd (Extended Data Fig. 4-2) at the EM level. This finding suggests that GABAergic neurons in the caudal GPe may exert an inhibitory modulation on the temporal cortices and lateral amygdala by regulating the activity of CR-expressing neurons in the MGd and PIN/PP. Since GABAergic neurons in the caudal GPe directly project to the temporal cortices (Fig. 2*C*), they may suppress the temporal cortices via two pathways: the direct pallido-cortical pathway and the indirect pallido–thalamo–cortical pathway.

10.1523/ENEURO.0161-24.2024.f4-1 Figure 4-1 CR-immunoreactive neurons projecting to the temporal cortices. FG was injected into the temporal association area. Almost all FG-labeled neurons exhibited CR immunoreactivity. Scale bar = 500 µm. Download Figure 4-1, TIF file.

10.1523/ENEURO.0161-24.2024.f4-2 Figure 4-2 GPe GABAergic fibers forming synaptic contacts on CR-expressing neurons in the MGd. (A) CR-immunoreactive neurons in MGd and PIN/PP were surrounded by numerous GFP-expressing GPe GABAergic fibers. (A’) Higher magnification from the rectangle in (A). (A’’) GFP and CR immunoreactivities were developed by using DAB-nickel and DAB, respectively. (B) An EM image shows the axon terminal (arrowhead) corresponding to the arrowheads in (A’, A”). (C) The GFP-immunoreactive axon terminal formed a symmetrical synaptic contact on the CR-immunoreactive soma (arrows). Scale bars = 200 µm in A, 20 µm in A’ and A”, 1 µm in B, and 0.2 µm in C. Download Figure 4-2, TIF file.

**Figure 5.**
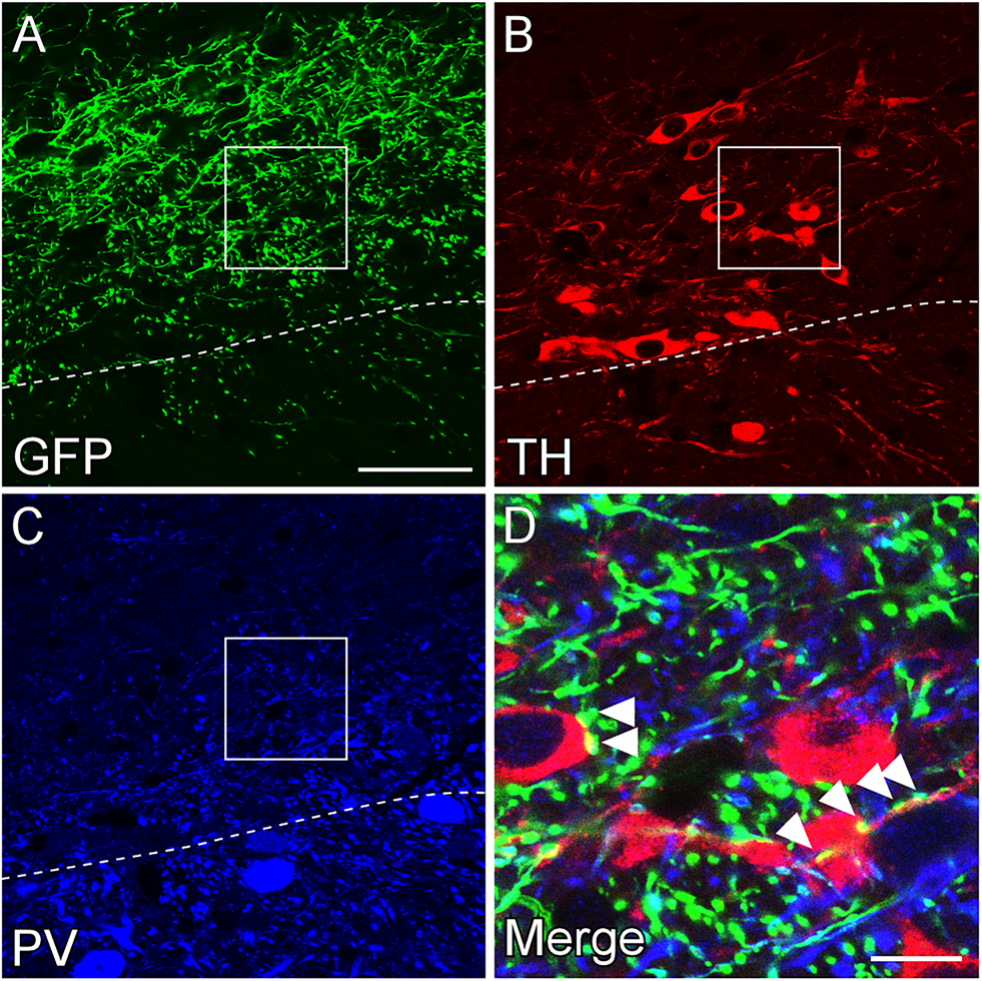
Axonal fibers of GPe GABAergic neurons in the SNL. ***A–C***, GFP-positive fibers were mainly distributed in the TH-positive region of SNL. The dashed line indicates the boundary between TH-positive and PV-positive regions. ***D***, The enlarged image of the area marked by the rectangle of ***A***. GFP-positive fibers form close synaptic apposition with TH-positive soma and dendrites (arrowheads). Scale bars: 50 µm in ***A*** and 10 µm in ***D***. See Extended Data Figure 5-1 for more details.

Axonal fibers originating from GABAergic neurons in the rostral GPe have previously been observed in the substantia nigra (Kita and Kita, 1994; Bolam et al., 2000; Mallet et al., 2012; Oh et al., 2017). To extend these observations and examine whether GPe GABAergic neurons project to dopaminergic or GABAergic neurons in the substantia nigra, we examined the distribution of GFP-immunoreactive axonal fibers from the caudal GPe using neurochemical markers of the substantia nigra, TH for dopaminergic neurons and PV for GABAergic neurons (Fig. 5; Extended Data Fig. 5-1; 98% of PV-positive neurons are GFP-positive neurons in the SNL of GAD67GFP mice, while none are TH-positive [122 PV-positive neurons in four animals)]. The boundary between TH- and PV-immunoreactive regions was distinct in the SNL. Most GFP-immunoreactive fibers were distributed in the TH-immunoreactive region and were in close contact with the TH-immunoreactive somata and dendrites. Only a few GFP-immunoreactive fibers were found in the PV-immunoreactive region. These findings suggest that GABAergic neurons in the caudal GPe may preferentially project to dopaminergic neurons in the SNL, consistent with the projection pattern of the rostral GPe to the substantia nigra par compacta (SNc; Oh et al., 2017).

10.1523/ENEURO.0161-24.2024.f5-1 Figure 5-1 Neurochemical profiles in the substantia nigra. (A) GAD67-GFP mice were used in this experiment. Lower magnification of the substantia nigra. (B) Higher magnification from the rectangle in (A). TH-immunoreactive neurons were negative for GFP (arrow), whereas PV-immunoreactive neurons were positive for GFP (arrowhead). (C) Higher magnification from the rectangle in (B). We confirmed the dopaminergic and GABAergic neurons using TH and PV immunoreactivity, respectively. Scale bars = 500 µm in A, 100 µm in B, and 10 µm in C. Download Figure 5-1, TIF file.

### Neurochemical properties in the caudal GPe

**Figure 6.**
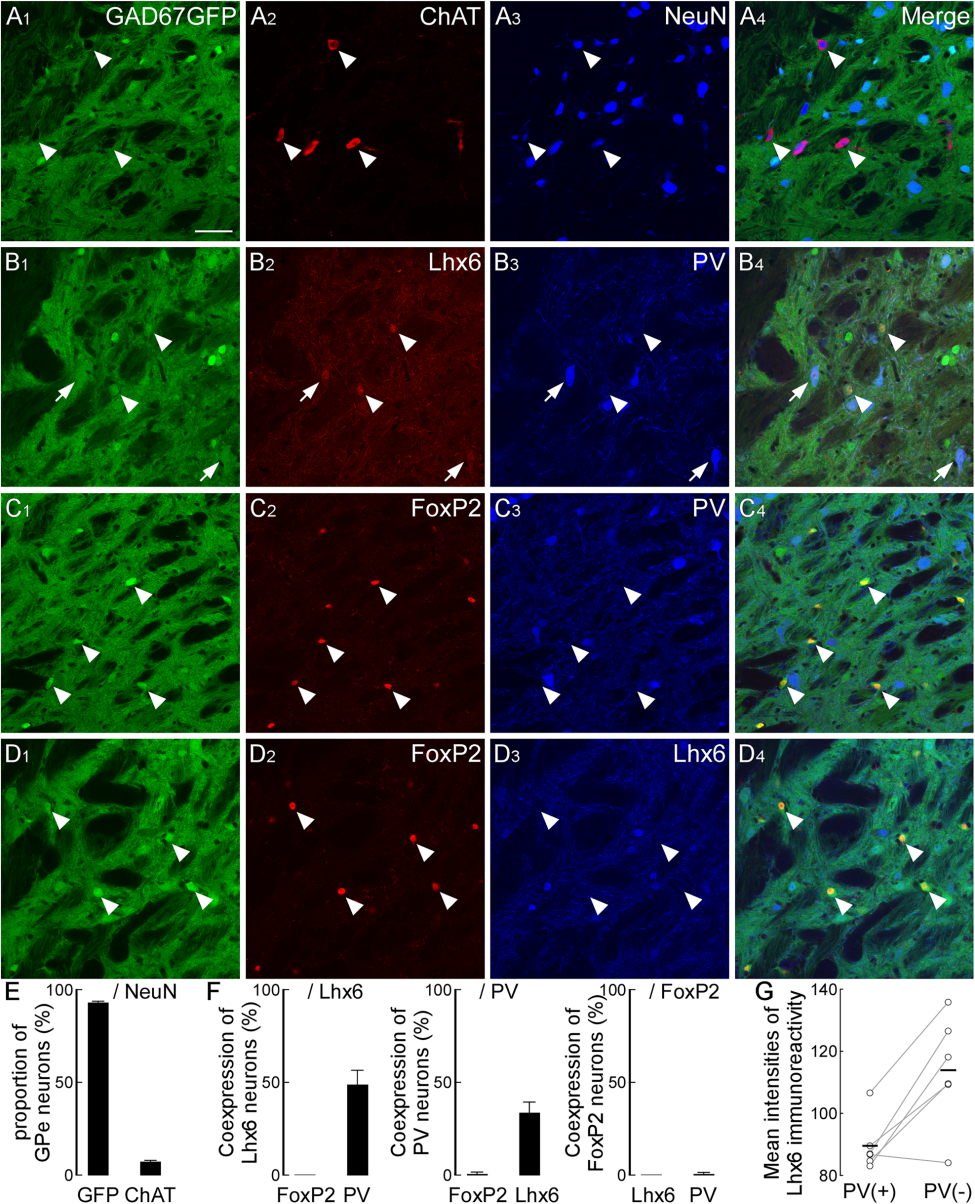
Neurochemical profiles in the caudal GPe. ***A***, GABAeric neurons express GFP in the GAD67-GFP mouse. While the somata of neurons strongly expressing GFP could be identified, somata of most GFP-positive neurons were not clearly identified. In contrast, ChAT-positive cholinergic neurons were completely negative for GFP (arrowheads). ***B***, Some of Lhx6- and GFP-positive neurons were immunoreactive for PV (arrow), but most of them were negative for PV (arrowheads). ***C***, FoxP2-positive neurons exhibited strongly GFP (arrowheads). Almost all of them were negative for PV. ***D***, All of FoxP2-positive neurons were negative for Lhx6 (arrowheads). ***E***, The vast majority of GPe neurons was GABAergic neurons. ***F***, Lhx6-immunoreactive neurons were colocalized with PV immunoreactivity. In contrast, FoxP2-immunoreactive neurons were not colocalized with Lhx6- and PV-immunoreactivities. ***G***, Among Lhx6-immunoreactive neurons, PV-positive neurons exhibited significantly weaker Lhx6 immunoreactivity than PV-negative neurons (*p < 0.05). Scale bar, 50 µm.

Recent studies have identified several GABAergic subpopulations in the rostral GPe, where distinct subpopulations characterized by neurochemical profiles exhibit specific projection targets (Mallet et al., 2012; Mastro et al., 2014; Abdi et al., 2015; Hernández et al., 2015; Oh et al., 2017; Abecassis et al., 2020). We hypothesized that similar distinct GABAergic subpopulations exist in the caudal GPe and project to specific brain regions. To test this hypothesis, we first examined their heterogeneity in the caudal GPe by using neurochemical markers ChAT, FoxP2, Lhx6, and PV in GAD67-GFP mice. ChAT-immunoreactive neurons were sparsely distributed (7.2% of GPe neurons, *n* = 504 neurons in three animals) and negative for GFP (Fig. 6*A*,*E*). In contrast, we observed variations in GFP intensities among GPe GABAergic neurons. FoxP2-immunoreactive neurons exhibited relatively strong GFP expression compared with other GPe GABAergic neurons, allowing clear identification of cell bodies (Fig. 6*C*,*D*) and consistently negative for Lhx6 (Fig. 6*F*; all FoxP2+ neurons were negative for Lhx6, *n* = 154 neurons in three animals) or PV (only 1.4% of FoxP2+ neurons were positive for PV, *n* = 154 neurons in three animals). PV-immunoreactive neurons typically displayed relatively large somata, some of which coexpressed Lhx6 [Fig. 6*B*,*F*; Lhx6-positive neurons (*n* = 107) and PV-positive neurons (*n* = 186) in four animals]. Among Lhx6-immunoreactive neurons, PV-positive neurons exhibited weaker Lhx6 immunoreactivity than PV-negative neurons (Fig. 6*G*; *p* = 0.0131; two-tailed paired *t* test; 37 ± 6 neurons across six animals). Overall, these results indicate that FoxP2-expressing GPe GABAergic neurons constitute a distinct subpopulation. In contrast, Lhx6- or PV-expressing GPe GABAergic neurons would be heterogeneous subpopulations and be further subdivided into three subpopulations: Lhx6-single, PV-single, and Lhx6- and PV-double-expressing. These neurochemical profiles in the caudal GPe are consistent with previous reports in the rostral GPe (Abdi et al., 2015; Hernández et al., 2015; Oh et al., 2017; Abecassis et al., 2020).

**Figure 7.**
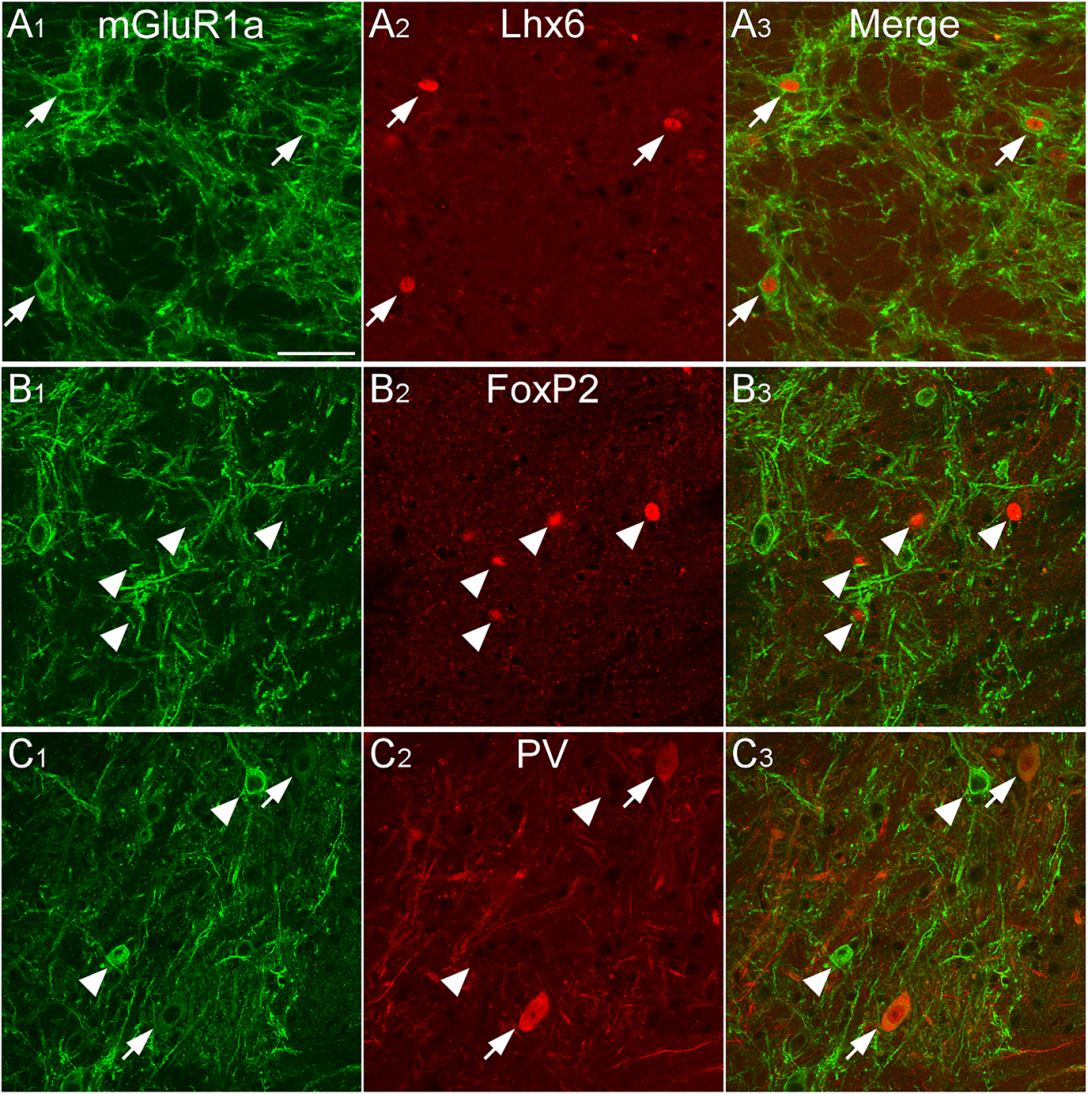
mGluR1a-immunoreactive neurons in the caudal GPe. mGluR1a immunoreactivity was mainly distributed in the dendrites and somata. ***A***, Most Lhx6-positive neurons exhibit strong immunoreactivity for mGluR1a (arrowheads). ***B***, FoxP2-positive neurons were not immunoreactive for mGluR1a (arrows). ***C***, Most PV-positive neurons show weak mGluR1a immunoreactivity (arrowheads). In contrast, strong mGluR1a-immunoreactive neurons were negative for PV immunoreactivity (arrows). Scale bar, 50 µm.

The GPe receives GABAergic input from the striatum and glutamatergic input mainly from subcortical regions and rarely from the cortex (Kaneko et al., 2002). Notably, Poisik et al. (2003) have reported the GABAergic subpopulations in the rostral GPe are subdivided by mGluR1-mediated depolarization. We next examined which GPe GABAergic subpopulations express mGluR1a. Immunoreactivity for mGluR1a was observed in the caudal GPe (Fig. 7), as previously reported in the rostral GPe. According to the intensity of mGluR1a immunoreactivity, two distinct subpopulations were observed: one with relatively strong mGluR1a immunoreactivity on the cell membrane, and another with weak mGluR1a expression. The strong mGluR1a-immunoreactive subpopulation was positive for Lhx6 (87.5% of strong mGluR1a+ neurons were positive for Lhx6; *n* = 40 in three animals) but negative for FoxP2 (0% of strong mGluR1a+ neurons were positive for FoxP2; *n* = 47 in three animals) and PV (0% of strong mGluR1a+ neurons were positive for PV; *n* = 33 in three animals). In contrast, the weak mGluR1a-immunoreactive subpopulation was positive for Lhx6 (97.5% of Lhx6a+ neurons were positive for weak mGluR1a; *n* = 161 in three animals) and PV (82.6% of PV+ neurons were positive for weak mGluR1a; *n* = 172 in three animals) but negative for FoxP2 (2.5% of FoxP2+ neurons were positive for weak mGluR1a; *n* = 197 in three animals). Overall, some Lhx6-expressing neurons showed strong mGluR1a immunoreactivity, while most Lhx6- or PV-expressing neurons exhibited weak mGluR1a immunoreactivity.

### GABAergic projecting neurons in the caudal GPe

**Figure 8.**
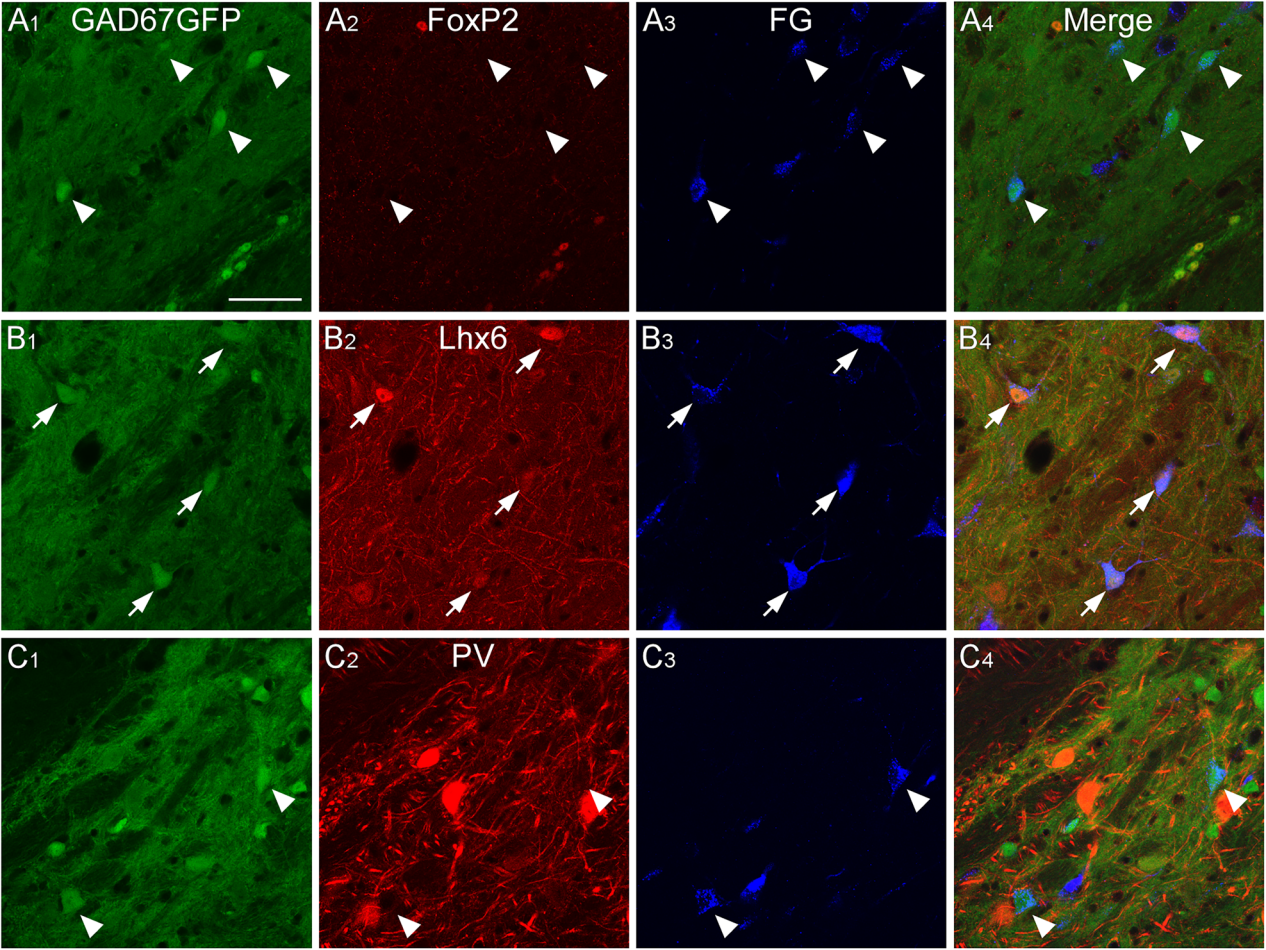
GABAergic neurons projecting to the temporal cortices in the caudal GPe. Injection of the retrograde tracer FG into the TeA of GAD67-GFP mice resulted in FG-labeled neurons in the caudal GPe. FG-labeled GABAergic neurons were positive for Lhx6 immunoreactivity (arrows in ***B***), but not for FoxP2 or PV (arrowheads in ***A*** and ***C***). Scale bar, 50  µm.

**Figure 9.**
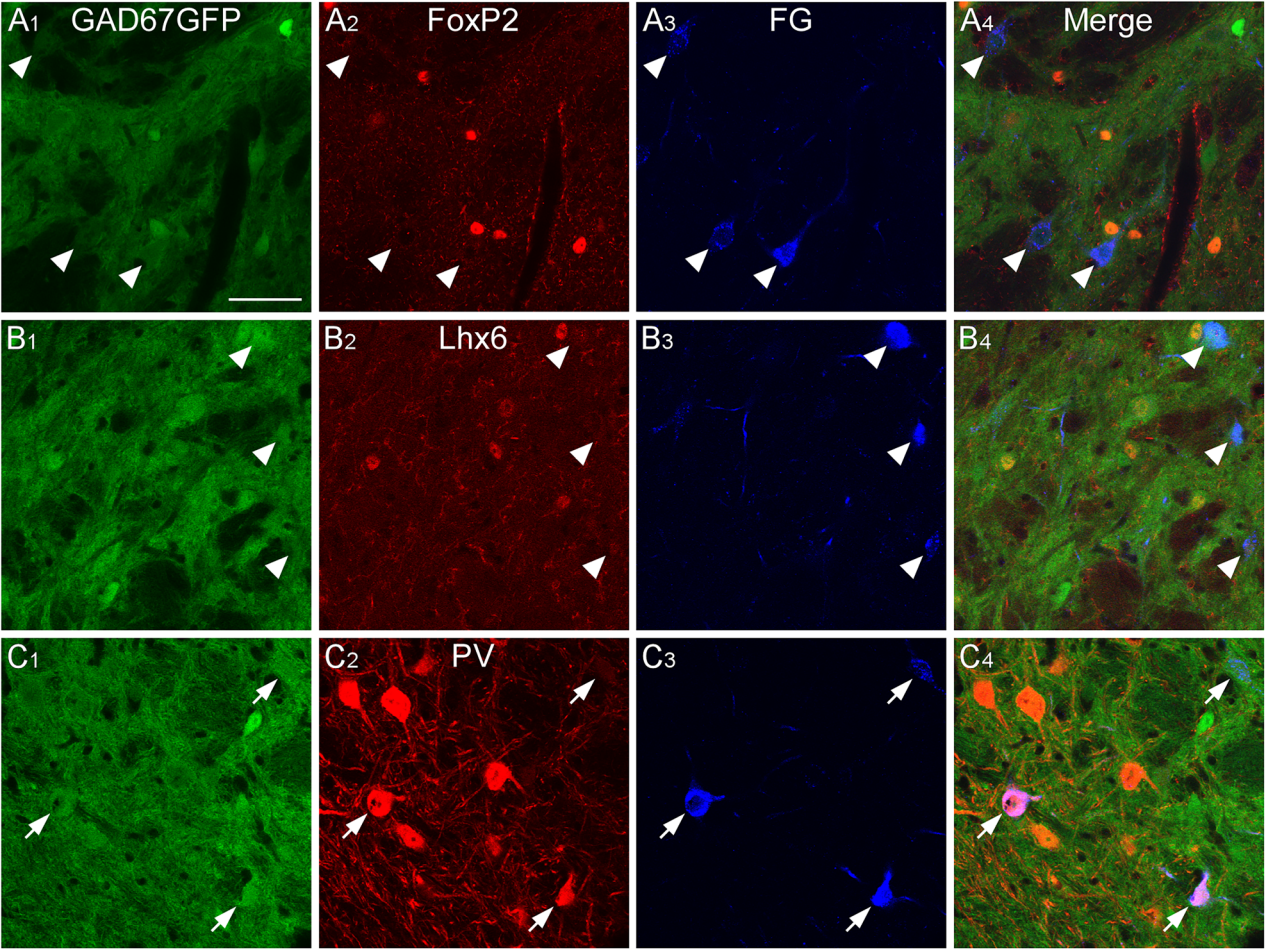
GABAergic neurons projecting to the MGB in the caudal GPe. Injection of FG into the MGB of GAD67-GFP mice resulted in FG-labeled neurons in the caudal GPe. FG-labeled GABAergic neurons were positive for PV immunoreactivity (arrows in ***C***), but not for FoxP2 or Lhx6 (arrowheads in ***A*** and ***B***). Scale bar, 50  µm.

**Figure 10.**
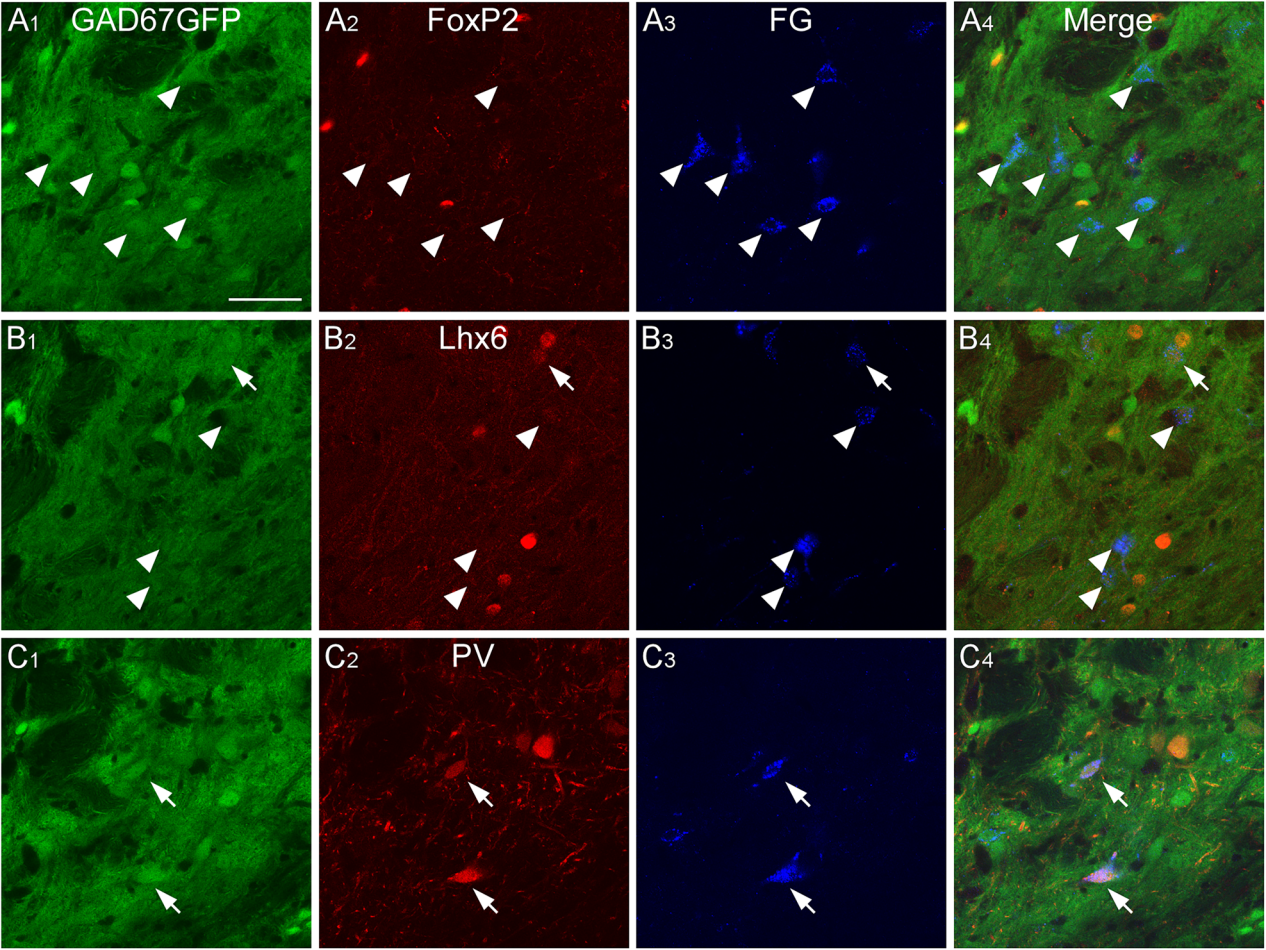
GABAergic neurons projecting to the CnF in the caudal GPe. Injection of FG into the CnF of GAD67-GFP mice resulted in FG-labeled neurons in the caudal GPe. Most FG-labeled GABAergic neurons were positive for PV immunoreactivity (arrows in ***C***), but not for FoxP2 or Lhx6 (arrowheads in ***A*** and ***B***). Scale bar, 50  µm.

**Figure 11.**
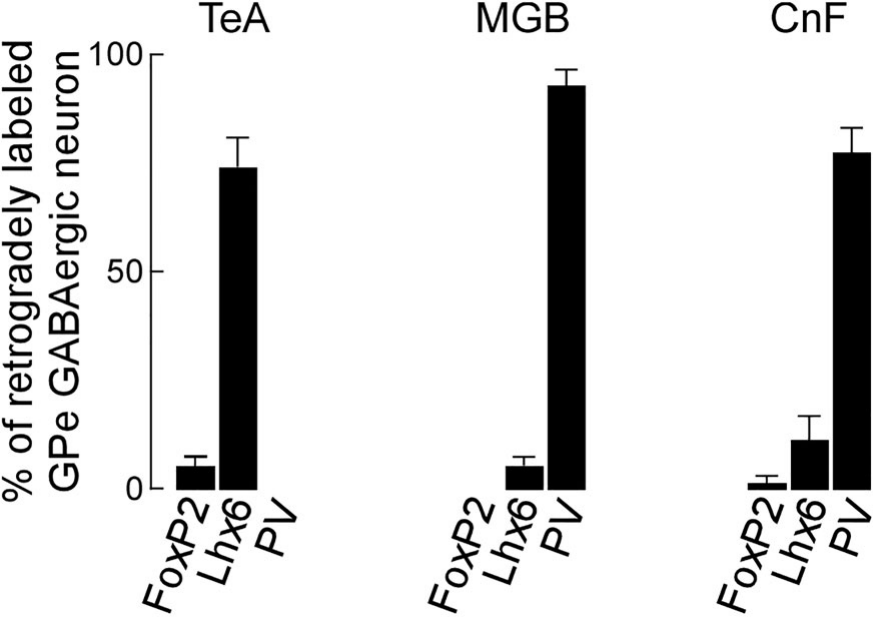
Summary of anatomical and neurochemical profiles in the caudal GPe. Lhx6-expressing neurons in the caudal GPe project into the TeA, whereas PV-expressing neurons into the MGB and CnF. See Extended Data Figure 11-1 for more details.

We showed that GABAergic neurons in the caudal GPe project to multiple brain regions and examined their heterogeneity using neurochemical profiles. We aimed to identify which subpopulations of GPe neurons project to specific regions. Based on our anterograde experiment (Fig. 2), we focused on three main projection areas: the TeA, MGB, and CnF. The neurochemical profiles of the retrogradely FG-labeled neurons in the caudal GPe were investigated using immunoreactivities for FoxP2, Lhx6, and PV. FG injection into the TeA demonstrated that most retrogradely labeled neurons were positive for Lhx6 in the caudal GPe (Figs. 8, 11; Extended Data Fig. 11-1*A*; 71.8% of FG- and GFP-double–labeled neurons exhibited Lhx6 immunoreactivity; *n* = 39 in four animals) but not for FoxP2 (5.8% of FG- and GFP-double–labeled neurons exhibited FoxP2 immunoreactivity; *n* = 52 in four animals) or PV (no FG- and GFP-double–labeled neurons exhibited PV immunoreactivity; *n* = 39 in four animals). FG injection into the MGB showed that most retrogradely labeled neurons were positive for PV (Figs. 9, 11; Extended Data Fig. 11-1*B*; 91.2% of FG- and GFP-double–labeled neurons exhibited PV immunoreactivity; *n* = 34 in four animals) but not for FoxP2 (No FG- and GFP-double–labeled neurons exhibited FoxP2 immunoreactivity; *n* = 91 in four animals) or for Lhx6 (3.7% of FG- and GFP-double–labeled neurons exhibited Lhx6 immunoreactivity; *n* = 54 in four animals). FG injection into the CnF showed that most retrogradely labeled neurons were positive for PV (Figs. 10, 11; Extended Data Fig. 11-1*C*; 75.4% of FG- and GFP-double–labeled neurons exhibited PV immunoreactivity; *n* = 114 in four animals), followed by Lhx6 (19.6% of FG- and GFP-double–labeled neurons exhibited Lhx6 immunoreactivity; *n* = 51 in four animals), but not for FoxP2 (no FG- and GFP-double–labeled neurons exhibited FoxP2 immunoreactivity; *n* = 54 in four animals). These observations suggest that Lhx6-expressing neurons are a distinct subpopulation projecting to the temporal cortices, whereas PV-expressing neurons project to the MGB and CnF. In addition, Lhx6- and PV-expressing neurons may project to the MGB and CnF, although this subpopulation may be minor.

10.1523/ENEURO.0161-24.2024.f11-1 Figure 11-1 The representative FG injection sites. The main injection sites were TeA (A), MGB (B), and CnF (C). Scale bar = 1.0 mm. Download Figure 11-1, TIF file.

### Presynaptic neurons of GABAergic neurons in the caudal GPe

**Figure 12.**
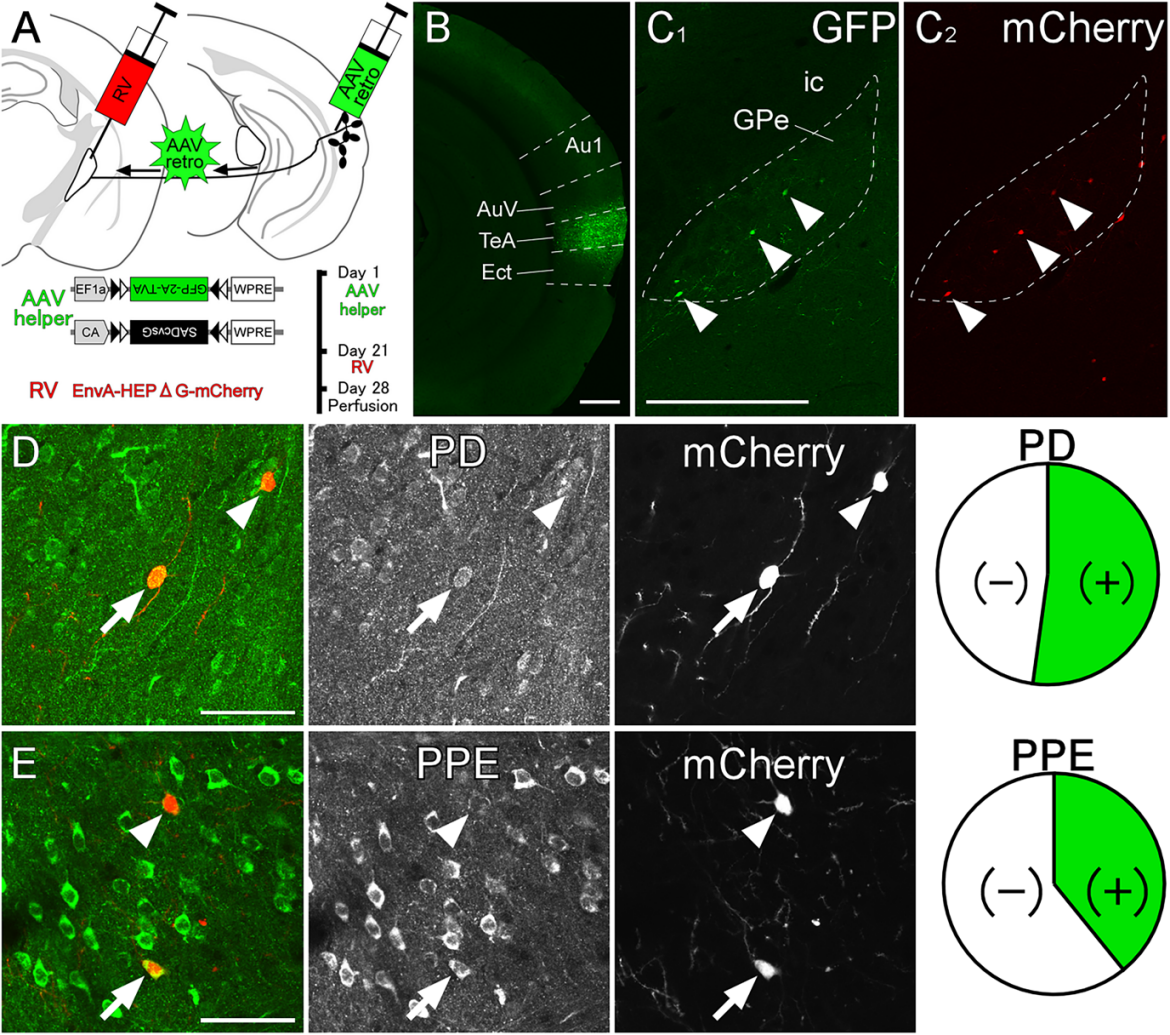
Inputs for GPe GABAergic neurons projecting to the auditory cortex. ***A***, The experimental schedule for (1) the injection of AAV helper virus, (2) the pseudotyped rabies virus (RV) injection, and (3) perfusion. ***B***, The injection site of AAV helper virus was mainly confined in the TeA. ***C***, The starter cells in the caudal GPe were identified by both GFP and mCherry expression (arrowheads). The dashed line indicates the caudal GPe. ***D, E***, Most of the transsynaptically labeled striatal neurons exhibited PD immunoreactivity (arrow), and some of them exhibited PPE immunoreactivity (arrow). Arrowheads indicate that mCherry-labeled neurons were negative for immunoreactivities. Scale bars, 0.5 mm in ***B*** and ***C***, 50 µm in ***D*** and ***E***. See Extended Data Figures 12-1 and 12-2 for more details.

We next investigated presynaptic neurons of GPe GABAergic neurons using the transsynaptic pseudotyped rabies virus expressing mCherry. To express the rabies glycoprotein and avian TVA receptor in GPe GABAergic neurons, we injected the AAV helper virus mixture into the TeA of VGAT-Cre mice (Fig. 12*A*). Three weeks later, we injected the pseudotyped rabies virus into the caudal GPe. Starter cells, which exhibited both GFP-immunoreactivity and mCherry fluorescence, were localized within the caudal GPe in three mice (Fig. 12*B*), as illustrated, and further analyzed. Transsynaptically mCherry-labeled presynaptic neurons were predominantly distributed in the TS, secondary in the caudal GPe and neocortex including the auditory cortex (Extended Data Fig. 12-1). The mCherry-labeled neurons in the TS were further examined using neurochemical markers to depict the neural circuit of the auditory cortico-basal ganglia loop. Neurons in the direct pathway express PD, whereas those in the indirect pathway express PPE (Le Moine et al., 1990; Lee et al., 1997; Sonomura et al., 2007). Most transsynaptically labeled presynaptic neurons were positive for PD (Fig. 12*C*; 52.2% of labeled neurons exhibited PD immunoreactivity; *n* = 115 in three animals), and some were positive for PPE (Fig. 12*D*; 39.2% of labeled neurons exhibited PPE immunoreactivity; *n* = 97 in three animals). Thus, GABAergic neurons in the caudal GPe that project into the TeA receive input from both direct and indirect pathway neurons in the TS. While previous anatomical studies have shown that both direct and indirect pathways project into the rostral GPe (Kawaguchi et al., 1990; Wu et al., 2000; Fujiyama et al., 2011), a recent physiological experiment in the rostral GPe has demonstrated that indirect pathway neurons project into FoxP2-negative prototypic neurons (Ketzef and Silberberg, 2021). This discrepancy may indicate a unique neural circuit in the posterior basal ganglia.

10.1523/ENEURO.0161-24.2024.f12-1 Figure 12-1 Distribution of presynaptic neurons of GPe GABAergic neurons. The parent neurons in the caudal GPe mainly received synaptic inputs from the TS. Hippocampus (Hippo), periaqueductal gray (PAG). Download Figure 12-1, TIF file.

10.1523/ENEURO.0161-24.2024.f12-2 Figure 12-2 Two control experiments for pseudotyped rabies virus. （A）AAVretro-CA-FLEX-SADcvsG and AAVretro-EF1a-FLEX-GFP-2A-TVA were injected into the primary auditory cortex of wild mice, following the injection of pseudotyped rabies virus into the same area. Although no GFP-labeled neuron was found, a few mCherry-labeled neurons were around the injection site in the three animals. (B) AAVretro-EF1a-FLEX-GFP-2A-TVA were injected into the temporal association cortex of VGAT-Cre mice, following the injection of pseudotyped rabies virus into the caudal part of GPe. This is due to confirm the distribution of starter cells. Immunofluorescence of GFP allowed visualization of even weak GFP signals. Almost all of mCherry-labeled neurons were distributed in the caudal GPe. Scale bars = 500 µm. Download Figure 12-2, TIF file.

Because it was reported that unpseudotyped rabies viruses would be introduced during virus preparation (Watabe-Uchida et al., 2012), we first tested whether the entire system functions in a Cre-dependent manner. AAVretro-CA-FLEX-SADcvsG and AAVretro-EF1a-FLEX-GFP-2A-TVA were injected into A1 of three wild mice, following the injection of pseudotyped rabies virus into the same area (Extended Data Fig. 12-2*A*). This resulted in no GFP-labeled neuron, a few mCherry-labeled neurons around the injection site (2 ± 2 neurons; mean ± SD among three mice), and no mCherry-labeled neurons outside the injection site, indicating this minimal labeling was likely caused by unavoidable contamination with the unpseudotyped rabies virus (Watabe-Uchida et al., 2012). Second, to confirm the distribution of starter cells, AAVretro-EF1a-FLEX-GFP-2A-TVA were injected into the temporal association cortex of VGAT-Cre mice, following the injection of pseudotyped rabies virus into the caudal part of GPe. Immunofluorescence of GFP allowed visualization of even weak GFP signals. Most of mCherry-labeled neurons were distributed in the caudal GPe (Extended Data Fig. 12-2*B*; 1 ± 1 neurons outside GPe and 18 ± 10 neurons in GPe; mean ± SD among four mice). GFP-immunoreactive neurons were observed in the caudal GPe, but not in the surrounding areas outside the GPe. Taken together, our method would primarily allow for the labeling of presynaptic neurons of GABAergic neurons in the caudal GPe.

## Discussion

**Figure 13.**
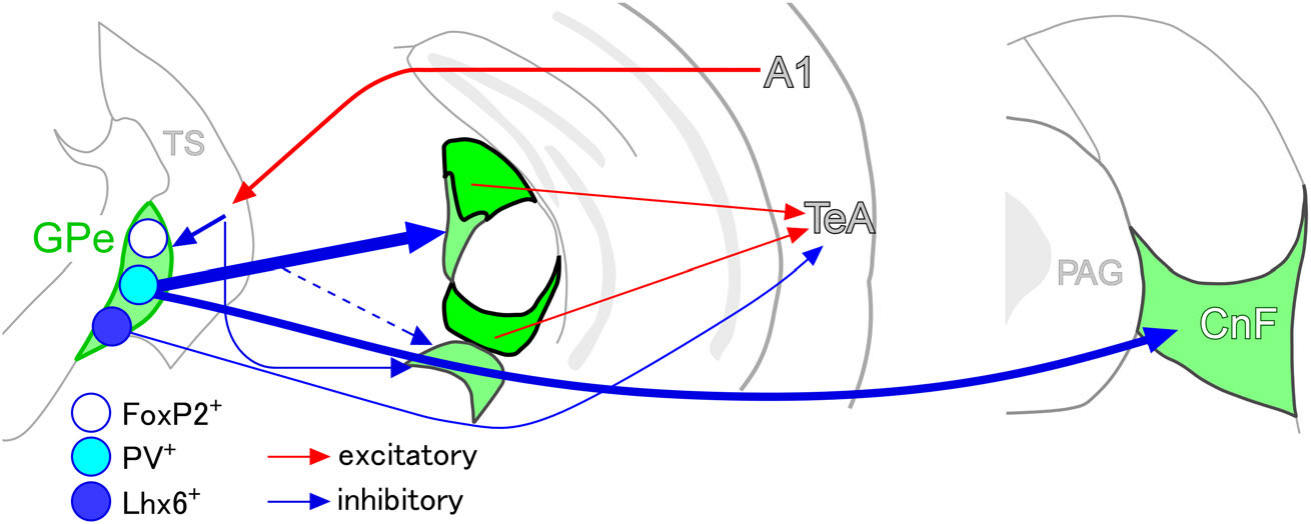
The schematic drawing of the auditory cortico-basal ganglia loop. The A1 sends excitatory efferents into the TS, where direct and indirect pathway neurons project into the caudal GPe. In the caudal GPe, Lhx6-expressing GABAergic neurons directly project to the temporal cortices, including the TeA/Ect/AuV, whereas PV-expressing GABAergic neurons strongly project to the CnF and MGB subdivisions, where CR-expressing neurons send excitatory efferents to the temporal cortices. It is worth noting that both Lhx6-expressing and PV-expressing neurons can directly and indirectly suppress the temporal cortices, respectively.

The present study contributes to elucidating the auditory cortico–basal ganglia loop in mice (Moriizumi and Hattori, 1992; Shammah-Lagnado et al., 1996; Hintiryan et al., 2016; Hunnicutt et al., 2016; Miyamoto et al., 2018; Cai et al., 2019; Foster et al., 2021; Valjent and Gangarossa, 2021). We first showed that the TS receives auditory input from A1and projects axonal fibers primarily to the caudal GPe. We further demonstrated that the caudal GPe relays to the temporal cortices via two pathways (Fig. 13). In the primary pathway, PV-expressing GABAergic neurons in the caudal GPe project to the nonlemniscal auditory thalamic nuclei, from where CR-expressing neurons subsequently relay to the temporal cortices (Extended Data Fig. 4-1; Cai et al., 2019; Belén Pardi et al., 2020). In the secondary pathway, Lhx6-expressing GABAergic neurons in the caudal GPe project directly to the temporal cortices. Both pathways of the caudal GPe exert an inhibitory influence on the nonlemniscal auditory pathway. Consequently, the caudal GPe emerged as the principal hub of the auditory cortico-basal ganglia loop. In addition, PV-expressing GABAergic neurons in the caudal GPe project primarily to the CnF and secondarily to dopaminergic neurons in the SNL and PAG. Interestingly, the GPi and SNR, which are considered to be the output nuclei in the basal ganglia, are not involved in the auditory cortico-basal ganglia loop.

Because feedforward projections from the A1 to the temporal cortices predominate over feedback projections (Vaudano et al., 1991; Tasaka et al., 2020), we propose that the auditory cortico-basal ganglia loop function as an open loop (Fig. 13; Joel and Weiner, 1994). Rather than functioning as a closed loop or feedback circuit, the auditory cortico-basal ganglia loop may act as a gate by spontaneously suppressing higher auditory cortices. It is because PV-expressing GABAergic neurons in the GPe, which may exhibit high-frequency spontaneous firing (Ketzef and Silberberg, 2021), could serve as another gate by suppressing the nonlemniscal auditory pathway. Additionally, Lhx6-expressing GABAergic neurons in the GPe could directly suppress the TeA. Thus, when acoustic stimuli activate the auditory cortex, the spontaneous inhibition of GPe GABAergic neurons may be relieved through disinhibition via the auditory cortico-striato-pallida pathway.

### Anatomical and neurochemical profiles of GABAergic neurons in the caudal GPe

As previously shown in the rostral GPe, the caudal GPe also consists of a large number of GABAergic neurons and a small number of cholinergic neurons (Fig. 6). Cholinergic neurons in the caudal GPe receive GABAergic input from the TS and project strongly to auditory cortices (Chavez and Zaborszky, 2017). Since the caudal GPe is close to the substantia innominata, cholinergic neurons in the caudal GPe may contribute to acetylcholine-dependent plasticity in the auditory cortex (Guo et al., 2019). From the neurochemical point of view, GPe GABAergic neurons in the caudal GPe can be divided into four major subpopulations. FoxP2-expressing neurons are a distinct subpopulation, as there is little colocalization with other neurochemical markers. Based on the colocalization of other neurochemical markers, we identified three other subpopulations: strong Lhx6-positive and PV-negative neurons; weak Lhx6-positive and PV-positive neurons; and Lhx6-negative and PV-positive neurons. From the anatomical point of view, GABAergic neurons in the caudal GPe can be divided into three major subpopulations. Lhx6-expressing GPe GABAergic neurons mainly projected to the temporal cortices, whereas PV-expressing GPe GABAergic neurons mainly projected to the MGB and CnF. However, in the present study, we were unable to ascertain the target region of FoxP2-expressing neurons, which might project to the striatum as arkypallidal neurons. Overall, our findings align with previous research in the rostral GPe, where distinct subpopulations characterized by neurochemical profiles exhibited specific projection targets (Mallet et al., 2012; Mastro et al., 2014; Abdi et al., 2015; Hernández et al., 2015; Oh et al., 2017; Abecassis et al., 2020). Interestingly, GABAergic neurons in the caudal GPe are likely similar to those in the rostral GPe based on anatomical and neurochemical profiles: PV-expressing neurons in both rostral and caudal GPe project to the thalamus (Mastro et al., 2014), while Lhx6-expressing neurons, which are nearly identical to Nkx2.1- and Npas4-positive neurons (Abecassis et al., 2020), project to the neocortex. It is important to note that the subregions targeted by GABAergic neurons from the rostral GPe differ from those targeted by neurons in the caudal GPe, as the rostral GPe would be integrated into the neural circuitry of distinct systems.

We also distinguished three distinct subpopulations expressing mGluR1a in the caudal GPe: subpopulations that strongly express mGluR1a with Lhx6, those that weakly express mGluR1a with Lhx6 or PV, and those that do not express mGluR1a with FoxP2. Previous electrophysiological studies have indicated that prototypic neurons are influenced by mGluR1a, whereas arkypallidal neurons are not, which aligns with our observations (Poisik et al., 2003). Kaneda et al. (2007) have reported a significant increase in the spontaneous firing rate in vitro with an mGluR1a agonist, suggesting its potential contribution to the sustained higher activity of prototypic neurons. Because the physiological significance of mGluR1a and specific sources of glutamatergic input remain unclear (Kaneko et al., 2002), further investigation is required.

### Functional implication of auditory cortico-basal ganglia loop

We demonstrated that the caudal GPe serves as the major output nucleus in the auditory basal ganglia. PV-expressing GPe GABAergic neurons and CR-expressing neurons in nonlemniscal MGB are involved in the cortico–striato–pallido–thalamo–cortical pathway, whereas Lhx6-expressing GPe GABAergic neurons participate in the cortico–striato–pallido-cortical pathway. Both pathways may suppress neuronal activity in the nonlemniscal auditory pathway. Our anatomical findings suggest that acoustic stimuli can activate neurons in higher auditory cortical areas by disinhibiting via those pathways. Indeed, nonlemniscal MGB and temporal cortices are known to respond to emotional sounds, including pup calls and threatening stimuli (Quirk et al., 1997; Dalmay et al., 2019; Barsy et al., 2020; Belén Pardi et al., 2020; Tasaka et al., 2020; Lee et al., 2021; Luo et al., 2022; Wang et al., 2023). CR-expressing neurons in the PIN/PP, which convey information regarding the association between a conditioned stimulus (neutral tone) and an unconditioned stimulus (footshock) to the lateral amygdala, have been implicated in triggering freezing behavior (Barsy et al., 2020; Belén Pardi et al., 2020). Considering the weak innervation of the central pathway of the auditory system from the caudal GPe, it is plausible that GABAergic neurons in the caudal GPe contribute more to emotional information than to auditory information processing.

We also demonstrated that GABAergic neurons in the caudal GPe directly innervate dopaminergic neurons in the SNL. Interestingly, dopaminergic neurons in the SNL primarily send axonal fibers to the TS (Menegas et al., 2018), suggesting the presence of a striato–pallido–nigral loop. This connection implies that the auditory cortico-basal ganglia loop shares information with dopaminergic neurons in the SNL that project to the TS, encode acoustic stimuli, and reinforce avoidance behavior in response to threatening stimuli and physical salience (Menegas et al., 2018; Akiti et al., 2022). Our anatomical findings suggest that acoustic stimuli can activate dopaminergic neurons in the SNL by disinhibition from the auditory cortico–striato–pallido–nigral pathway. Indeed, acoustic stimuli have been shown to activate dopaminergic neurons in the SNL (Menegas et al., 2018).

In addition, we demonstrated that PV-expressing GABAergic neurons in the caudal GPe project to the CnF. Considering that glutamatergic neurons in the CnF elicit high-speed, synchronous-gait locomotion (Caggiano et al., 2018; Dautan et al., 2021), the auditory GPe→CnF pathway might play an important role in locomotion, including escape-like behaviors (Li et al., 2021). Moreover, this pathway may serve as a substrate for the improvement of walking in patients with Parkinson’s disease using rhythmic beats (Nombela et al., 2013).

In summary, the caudal GPe is a principal hub in the auditory cortico-basal ganglia loop. The auditory cortico-basal ganglia loop has an inhibitory effect on the higher auditory thalamic and cortical regions and may be instrumental in defensive behaviors against acoustic stimuli.

## Synthesis

Reviewing Editor: Michael Michaelides, NIDA-NIH Decisions are customarily a result of the Reviewing Editor and the peer reviewers coming together and discussing their recommendations until a consensus is reached. When revisions are invited, a fact-based synthesis statement explaining their decision and outlining what is needed to prepare a revision will be listed below. The following reviewer(s) agreed to reveal their identity: Mitsuko Watabe-Uchida, Daniel Llano. Note: If this manuscript was transferred from JNeurosci and a decision was made to accept the manuscript without peer review, a brief statement to this effect will instead be what is listed below.

Reviewer 1

Tomioka et al. examined anatomical connectivity of caudal globus pallidus (GPe). Classically, it was known that the caudal GPe projects not only to the substantia nigra pars latelaris (SNL), the output nucleus of the basal ganglia, but also to other nuclei outside of the basal ganglia (Shammah-Lagnado et al., 1996). Consistent with Sahammah-Lagndo et al., authors found that the caudal GPe projects to the temporal cortex and medial geniculate body (MGB), in addition to the SNL. They also found several neuron types in the caudal GPe, consistent with the previous findings in the rostral GPe.

Overall, the molecular and anatomical profiles of the caudal GPe are useful. Some conclusions do not match with previous observations and should be rephrased. Some control experiments are missing.

Major concerns

1. Tracing with rabies virus requires several critical control experiments. First, to confirm that the entire system is working in a Cre-dependent manner, exactly the same combination of virus should be injected into control (Cre-) animals. Second, to confirm that the starter neurons are restricted in the caudal GPe, rabies virus and AAV-TVA should be injected without RG. Although authors checked double positive neurons for GFP and mCherry for this purpose, it is well known that detection threshold of GFP is much higher than required expression level of TVA for rabies infection. Third, injection loci in TeA should be labeled with a separate dye, beads or AAV. By the way, "PRV" means pseudorabies virus, which is totally different from modified rabies virus.

2. Although authors emphasize that their findings are different from classical concepts and findings in other striatum subareas, it is not clear what is unique. The popular classical model for cortico-basal ganglia loops consists of many loops (Alexander et al., 1986) and the same group added a loop through the temporal cortex and caudate tail (TS) (Middleton and Strick, 1996). Authors should frame their findings related to these studies. Further, the previous anatomy study of the caudal GPe by Shammah-Lagnado et al., 1996 should be cited more appropriately, because all the projection targets authors chose were reported in this previous study. In addition, because multiple studies reported various projection targets of the more anterior GPe, the difference (or similarity) of the caudal GPe inputs/targets should be clearly stated.

3. GPe projection to SNL, but not to SNr should not be interpreted as "distinct from the classical concept". SNL is the lateral-most extension of SNr and SNc. Further, the preferential projection of the caudal GPe to dopamine neurons in SNL should be quantified if authors want to claim so. As far as the Figure 5D shows, there are many blue PV neuron dendrites near green GPe axons. In addition, it has to be demonstrated whether all SNL GABA neurons are PV neurons.

4. For quantification, indicate the animal number, not only the cell number. Authors should first quantify within an animal, and then perform statistical test across animals.

Other points

• Because the caudal GPe is the main focus in this study, the location should be clearly defined.

• The definition of "strong" vs "weak" expression should be defined.

• Line 439: "GPe that project into the auditory cortex" should be project into the "TeA".

• Quantification of the axon fiber density should be described in the Method.

• Line 394: typo "mGlu1a"

Reviewer 2

The work titled "The External Globus Pallidus as the hub of the auditory cortico-basal ganglia loop" by Tomioka et al extensively examined the GABAergic cells of the caudal globus pallidus externus (Gpe) as a part of the auditory corticostriatal circuit. In this study, the authors showed the GABAergic cells of the caudal GPe project into different cortical and subcortical brain areas such as TeA, MGB, and cuneuform nucleus (CnF). These GABAergic cells were found to have heterogeneous neurochemical profiles, which could control the output of these cells to other brain regions. Using a rabies-virus-derived tracing approach, the GABAergic cells projecting to temporal association area (TeA) were found to receive inputs from other brain regions specifically tail of striatum (TS) and auditory cortex (AC).

Overall, this study provides important new details about basal ganglia circuits that process auditory information. The study is well-illustrated and manuscript is well-written. I have comments below that are intended to improve the impact of the paper.

Major:

- In Figure 1: Panel C does not clearly show the cell bodies of the postsynaptic corticostriatal neurons in the TS. Providing an enlarged image of those cells with DAPI labeling will be more appropriate. it is important to use DAPI or anti-NeuN as cellular or neuronal markers, respectively, to easily identify the soma. Also, it will be more appropriate to label the fiber ending with a vascular marker or axonal marker. Additionally, I do not know the significance of panel E2, which shows the label against TH (I guess it is Tyrosine hydroxylase). The description of panel E2 is not mentioned in the figure's legend.

- The authors argued in the introduction about the possible role of the cortico-basal ganglia loop in sensory processing by addressing the input projections from the sensory cortex to the basal ganglia. Still, most of the work addresses the universal output projections of the GABAergic cells of the globus pallidus. It would be better to address how the efferents of GPe function in sensory processing.

Minor:

- The author should mention the brain atlas they followed.

- How the boundaries of MGB were determined by PKCδ immunoreactivity. I think the authors should add a supporting reference for the statement mentioned in lines 262-263.

- The coordinates given to TeA did not show the lateral coordinates. Were these injections made directly from the surface of the brain's side? If yes, please provide the angle to the horizontal line the authors used to target the surface.

- Line 328: Please add a supporting reference.

- Figure 6: Which statistical tests have been used? How many animals are used? Also how many cells have been counted per animal and in total? Please mention the detailed tests.

There is no labeling of the brain regions in the extended Figure 6.

## Author Response

Dear Dr. Michael Michaelides and Reviewers, We would like to express our sincere gratitude to you and the reviewers for the time and effort spent reviewing our manuscript. We highly appreciate the valuable comments and suggestions, which have greatly helped us improve the quality of our work.

We have carefully addressed each comment and made the necessary revisions. Below, we provide a point-by-point response to the reviewers' comments. Each comment is followed by our response, and the corresponding changes in the manuscript are highlighted in the revised version.

Reviewer 1:

Major concerns 1. Tracing with rabies virus requires several critical control experiments. First, to confirm that the entire system is working in a Cre-dependent manner, exactly the same combination of virus should be injected into control (Cre-) animals. Second, to confirm that the starter neurons are restricted in the caudal GPe, rabies virus and AAV-TVA should be injected without RG. Although authors checked double positive neurons for GFP and mCherry for this purpose, it is well known that detection threshold of GFP is much higher than required expression level of TVA for rabies infection. Third, injection loci in TeA should be labeled with a separate dye, beads or AAV. By the way, "PRV" means pseudorabies virus, which is totally different from modified rabies virus.

We thank the reviewer for this insightful comment. To address the concerns regarding the pseudotyped rabies virus, we have conducted additional control experiments.

1) We injected the same AAV and pseudotyped rabies viruses into the primary auditory cortex of wild-type mice on the same schedule as the previous one. As a result, we confirmed a small number of mCherry-positive pyramidal neurons (2 {plus minus} 2 neurons; mean {plus minus} SD among three mice), indicating this minimal labeling was likely caused by unavoidable contamination with the unpseudotyped rabies virus (Watabe-Uchida et al., 2012). We have prepared Figure 12-2 to illustrate this result.

2) We injected AAVretro-EF1a-FLEX-GFP-2A-TVA into TeA and the pseudotyped rabies virus into the GPe on the same schedule as the previous one. As a result, we confirmed that nearly all mCherry-labeled neurons exhibited GFP-immunoreactivity and most of mCherry-labeled neurons were distributed in the caudal GPe (Fig. 12-2B; 1 {plus minus} 1 neurons outside GPe and 18 {plus minus} 10 neurons in GPe; mean {plus minus} SD among four mice). We have prepared Fig. 12-2 to illustrate this result.

3) Since we injected AAVretro-EF1a-FLEX-GFP-2A-TVA into the TeA, we can confirm the injection site by GFP expression. We have added the image of injection site in the Figure 12.

4) We have replaced PRV with pseudotyped rabies virus in the revised manuscript.

We have added the following sentences in the revised manuscript.

Lines 149-156 in the materials and methods "We performed two control experiments using pseudotyped rabies virus. To confirm the contamination of unpseudotyped rabies virus in vivo, AAVretro-CA-FLEX-SADcvsG and AAVretro-EF1a-FLEX-GFP-2A-TVA were injected into the primary auditory cortex of C57BL/6J mice, following the injection of pseudotyped rabies virus into the same area. In another control experiment, to confirm the distribution of starter cells, AAVretro-EF1a-FLEX-GFP-2A-TVA were injected into the temporal association cortex of VGAT-Cre mice, following the injection of pseudotyped rabies virus into the caudal GPe." Lines 409-411 in the results "Starter cells, which exhibited both GFP-immunoreactivity and mCherry fluorescent, were localized within the caudal GPe in three mice (Fig. 12B), as illustrated, and further analyzed." Lines 429-446 in the results "Because it was reported that unpseudotyped rabies viruses would be introduced during virus preparation (Watabe-Uchida et al., 2012), we first tested whether the entire system functions in a Cre-dependent manner. AAVretro-CA-FLEX-SADcvsG and AAVretro-EF1a-FLEX-GFP-2A-TVA were injected into the primary auditory cortex of three wild mice, following the injection of pseudotyped rabies virus into the same area (Fig. 12-2A). This resulted in no GFP-labeled neuron, a few mCherry-labeled neurons around the injection site (2 {plus minus} 2 neurons; mean {plus minus} SD among three mice), and no mCherry-labeled neurons outside the injection site, indicating this minimal labeling was likely caused by unavoidable contamination with the unpseudotyped rabies virus (Watabe-Uchida et al., 2012). Secondly, to confirm the distribution of starter cells, AAVretro-EF1a-FLEX-GFP-2A-TVA were injected into the temporal association cortex of VGAT-Cre mice, following the injection of pseudotyped rabies virus into the caudal part of GPe. Immunofluorescence of GFP allowed visualization of even weak GFP signals. Most of mCherry-labeled neurons were distributed in the caudal GPe (Fig. 12-2B; 1 {plus minus} 1 neurons outside GPe and 18 {plus minus} 10 neurons in GPe; mean {plus minus} SD among four mice). GFP-immunoreactive neurons were observed in the caudal GPe, but not in the surrounding areas outside the GPe. Taken together, our method would primarily allow for the labeling of presynaptic neurons of GABAergic neurons in the caudal GPe." 2. Although authors emphasize that their findings are different from classical concepts and findings in other striatum subareas, it is not clear what is unique. The popular classical model for cortico-basal ganglia loops consists of many loops (Alexander et al., 1986) and the same group added a loop through the temporal cortex and caudate tail (TS) (Middleton and Strick, 1996). Authors should frame their findings related to these studies. Further, the previous anatomy study of the caudal GPe by Shammah-Lagnado et al., 1996 should be cited more appropriately, because all the projection targets authors chose were reported in this previous study. In addition, because multiple studies reported various projection targets of the more anterior GPe, the difference (or similarity) of the caudal GPe inputs/targets should be clearly stated.

We thank the reviewer for this insightful comment. We have added the following sentences in the revised manuscript.

Lines 43-56 in the introduction for Alexander et al., (1986) "Alexander et al., (1986) proposed five cortico-basal ganglia loops: motor, oculomotor, dorsolateral prefrontal, lateral orbitofrontal, and anterior cingulate gyrus. This model was later developed and conceptualized to process three types of information: motor, limbic, and associative (Joel and Weiner, 1994; Kompoliti and Metman, 2010; Simonyan, 2019). In this framework, the motor and oculomotor loops in the Alexander's model are categorized as motor, the dorsolateral prefrontal and lateral orbitofrontal loops as associative, and the anterior cingulate loop as limbic. In the context of cortico-basal ganglia loops for sensory inputs, Alexander et al. (1986) documented somatosensory inputs within the framework of the motor loop, while Middleton and Strick (1996) demonstrated a cortico-basal ganglia loop associated with higher-order visual areas in primates, involving the cortico-strio-nigro-thalamo-cortical loop circuit, using herpes simplex virus type 1. Despite these findings, descriptions of cortico-basal ganglia loops for sensory modalities remain limited, with no detailed descriptions related to auditory processing." Lines 272-277 in the results for Middleton and Strick (1996) "Our anatomical evidence aligns with the previous study using PHA-L tracing (Shammah-Lagnado et al., 1996), which demonstrated that GPe GABAergic neurons project to the lateral hypothalamus and substantia nigra pars lateralis using a retrograde tracer. Thus, the present study is the first to demonstrate that GPe GABAergic neurons also project to the cuneiform nucleus and cortical areas." Lines 499-506 in the discussion for the difference (or similarity) "Interestingly, GABAergic neurons in the caudal GPe are likely similar to those in the rostral GPe based on anatomical and neurochemical profiles: PV-expressing neurons in both rostral and caudal GPe project to the thalamus (Mastro et al., 2014), while Lhx6-expressing neurons, which are nearly identical to Nkx2.1- and Npas4-positive neurons (Abecassis et al., 2020), project to the neocortex. It is important to note that the subregions targeted by GABAergic neurons from the rostral GPe differ from those targeted by neurons in the caudal GPe, as the rostral GPe would be integrated into the neural circuitry of distinct systems." 3. GPe projection to SNL, but not to SNr should not be interpreted as "distinct from the classical concept". SNL is the lateral-most extension of SNr and SNc. Further, the preferential projection of the caudal GPe to dopamine neurons in SNL should be quantified if authors want to claim so. As far as the Figure 5D shows, there are many blue PV neuron dendrites near green GPe axons. In addition, it has to be demonstrated whether all SNL GABA neurons are PV neurons.

We agree with the reviewer's comment: SNL is the lateral-most extension of SNr and SNc. Thus, we have deleted those lines in the revised manuscript: "distinct from the classical concept" Regarding the preferential projection of the caudal GPe to the SNL, we first examined the colocalization of PV immunoreactivity and GFP fluorescence in the SNL of GAD67-GFP mice. As a result, 98% of PV-positive neurons are GABAergic neurons. Thus, we have added the following sentences in the revised manuscript.

Lines 318-323 in the results "we examined the distribution of GFP-immunoreactive axonal fibers from the caudal GPe using neurochemical markers of the substantia nigra, tyrosine hydroxylase (TH) for dopaminergic neurons and parvalbumin (PV) for GABAergic neurons (Fig. 5 and Fig. 5-1; 98% of PV-positive neurons are GFP-positive neurons in the SNL of GAD67GFP mice, while none are TH-positive (122 PV-positive neurons in 4 animals))." We have softened the tone of our argument to account for the numerous uncertainties regarding the synaptic connections from GPe GABAergic neurons to the SNL.

Lines 326-329 in the results "These findings suggest that GABAergic neurons in the caudal GPe may preferentially project to dopaminergic neurons in the SNL, consistent with the projection pattern of the rostral GPe to the SNc (Oh et al., 2017)." 4. For quantification, indicate the animal number, not only the cell number. Authors should first quantify within an animal, and then perform statistical test across animals.

We have followed the reviewer's suggestion in the revised manuscript.

Lines 338-349 in the results "ChAT-immunoreactive neurons were sparsely distributed (7.2% of GPe neurons: n = 504 neurons in three animals) and negative for GFP (Fig. 6A, E). In contrast, we observed variations in GFP intensities among GPe GABAergic neurons. FoxP2-immunoreactive neurons exhibited relatively strong GFP expression compared with other GPe GABAergic neurons, allowing clear identification of cell bodies (Fig. 6C, D) and consistently negative for Lhx6 (Fig. 6F; All FoxP2+ neurons were negative for Lhx6, n = 154 neurons in 3 animals) or PV (only 1.4% of FoxP2+ neurons were positive for PV, n = 154 neurons in 3 animals). PV-immunoreactive neurons typically displayed relatively large somata, some of which co-expressed Lhx6 (Fig. 6B, F; Lhx6-positive neurons (n = 107) and PV-positive neurons (n = 186) in four animals). Among Lhx6-immunoreactive neurons, PV-positive neurons exhibited weaker Lhx6 immunoreactivity than PV-negative neurons (Fig. 6G; p = 0.0131, two-tailed paired t-test, 37 {plus minus} 6 neurons across 6 animals)." Lines 364-373 in the results "The strong mGluR1a-immunoreactive subpopulation was positive for Lhx6 (87.5% of strong mGluR1a+ neurons were positive for Lhx6, n = 40 in three animals), but negative for FoxP2 (0% of strong mGluR1a+ neurons were positive for FoxP2, n = 47 in three animals) and PV (0% of strong mGluR1a+ neurons were positive for PV, n = 33 in three animals). In contrast, the weak mGluR1a-immunoreactive subpopulation was positive for Lhx6 (97.5% of Lhx6a+ neurons were positive for weak mGluR1a, n = 161 in three animals) and PV (82.6% of PV+ neurons were positive for weak mGluR1a, n = 172 in three animals), but negative for FoxP2 (2.5% of FoxP2+ neurons were positive for weak mGluR1a, n = 197 in three animals)." Lines 382-399 in the results "FG injection into the TeA demonstrated that most retrogradely labeled neurons were positive for Lhx6 in the caudal GPe (Figs. 8 and 11 and Fig. 11-1A; 71.8% of FG- and GFP-double-labeled neurons exhibited Lhx6 immunoreactivity. n = 39 in four animals) but not for FoxP2 (5.8% of FG- and GFP-double-labeled neurons exhibited FoxP2 immunoreactivity. n = 52 in four animals) or PV (No FG- and GFP-double-labeled neurons exhibited PV immunoreactivity. n = 39 in four animals). FG injection into the MGB showed that most retrogradely labeled neurons were positive for PV (Figs. 9 and 11 and Fig. 11-1B; 91.2% of FG- and GFP-double labeled neurons exhibited PV immunoreactivity. n = 34 in four animals) but not for FoxP2 (No FG- and GFP-double labeled neurons exhibited FoxP2 immunoreactivity. n = 91 in four animals), or for Lhx6 (3.7% of FG- and GFP-double labeled neurons exhibited Lhx6 immunoreactivity. n = 54 in four animals). FG injection into the CnF showed that most retrogradely labeled neurons were positive for PV (Figs. 10 and 11 and Fig. 11-1C; 75.4% of FG- and GFP-double-labeled neurons exhibited PV immunoreactivity. n = 114 in four animals), followed by Lhx6 (19.6% of FG- and GFP-double-labelled neurons exhibited Lhx6 immunoreactivity. n = 51 in four animals), but not for FoxP2 (no FG- and GFP-double-labeled neurons exhibited FoxP2 immunoreactivity. n = 54 in four animals)." Other points • Because the caudal GPe is the main focus in this study, the location should be clearly defined.

Lines 257-259 in the results "we extensively analyzed the neural circuit via the caudal GPe (-1.06 to -1.7 mm from Bregma along the rostrocaudal axis) in the present study." • The definition of "strong" vs "weak" expression should be defined.

Those terms were used to compare GFP fluorescent or neurochemical immunoreactivities among two or three subpopulations. In the revised manuscript, we have added additional description to ensure greater clarity.

Lines 340-342 in the results "FoxP2-immunoreactive neurons exhibited relatively strong GFP expression compared with other GPe GABAergic neurons, allowing clear identification of cell bodies (Fig. 6C, D)" Lines 361-364 in the results "According to the intensity of mGluR1a immunoreactivity, two distinct subpopulations were observed: one with relatively strong mGluR1a immunoreactivity on the cell membrane, and another with weak mGluR1a expression." • Line 439: "GPe that project into the auditory cortex" should be project into the "TeA".

We have corrected it. • Quantification of the axon fiber density should be described in the Method.

There is the description in the materials and methods.

Lines 222-234 in the materials and methods "Five mice were used for the quantitative measurements of axonal fibers. Image analysis was performed using a previously described method, with slight modifications (Tomioka et al., 2023). Similar coronal sections were analyzed for the quantitative measurement of GFP-labeled axonal fibers from the GPe. Images were obtained using a confocal laser-scanning microscope (FV3000) with a 10× objective lens at a 2048 × 2048 pixel resolution. All images were analyzed using MATLAB software (R2019b; MathWorks, MA, USA). Briefly, the boundaries of the MGB subdivisions were defined according to PKCδ immunoreactivity (Tomioka et al., 2023). The area of each subdivision was calculated. The raw images of GFP-positive signals were converted into binary images using one threshold per section. The summed area of GFP-positive signals in each subdivision was calculated. Finally, the occupancy of GFP-positive signals in each MGB subdivision was computed. Friedman's test and Bonferroni's post hoc test were used for multiple comparisons." • Line 394: typo "mGlu1a" We have corrected it.

Reviewer 2:

Major:

- In Figure 1: Panel C does not clearly show the cell bodies of the postsynaptic corticostriatal neurons in the TS. Providing an enlarged image of those cells with DAPI labeling will be more appropriate. It is important to use DAPI or anti-NeuN as cellular or neuronal markers, respectively, to easily identify the soma. Also, it will be more appropriate to label the fiber ending with a vascular marker or axonal marker. Additionally, I do not know the significance of panel E2, which shows the label against TH (I guess it is Tyrosine hydroxylase). The description of panel E2 is not mentioned in the figure's legend.

We thank the reviewer for this insightful comment. We have revised Figure 1 B and C. In Figure1B, tdTomato-positive neurons were illustrated with NeuN immunoreactivity. In Figure 1C, tdTomato-positive fibers were illustrated with synaptophysin immunoreactivity. As for Figure 1E, Tyrosine hydroxylase (TH) immunoreactivity was used to separate subregions of the substantia nigra.

We have included the following descriptions in the figure legend for panels B-E.

Lines 775-783 in the legends "(B1) AAV2-CAG-FLEX-tdTomato was injected into the tail of striatum (TS). (B2-B4) The enlarged image of the area marked by the rectangle of (B1). The tdTomato-expressing neurons were found within the TS. (C) Dense tdTomato-positive striatal fibers were in the caudal GPe. (D1) The enlarged image of the area marked by the rectangle of (C). The distribution of tdTomato-positive axon terminals was similar to that of synaptophysin immunoreactivity in the caudal GPe (D2). (E1) tdTomato-positive striatal fibers were in the substantia nigra par lateralis (SNL), but not in the substantia nigra par compacta (SNC) or reticulata (SNR). (E2) Tyrosine hydroxylase (TH)-immunoreactivity was used to define the boundaries of the substantia nigra." - The authors argued in the introduction about the possible role of the cortico-basal ganglia loop in sensory processing by addressing the input projections from the sensory cortex to the basal ganglia. Still, most of the work addresses the universal output projections of the GABAergic cells of the globus pallidus. It would be better to address how the efferents of GPe function in sensory processing.

We appreciate the reviewer's insightful comment. Noticing a gap between the introduction and discussion, we have revised both sections in line with the reviewer's suggestions.

Lines 35-41 in the introduction "It has become evident that GABAergic neurons in the rostral GPe represent a heterogeneous population, with distinct subgroups defined by their gene expression profiles and projection targets (Bolam et al., 2000; Mallet et al., 2012; Abdi et al., 2015; Oh et al., 2017; Abecassis et al., 2020). Notably, some GPe neurons project to the thalamus and cortex (Mastro et al., 2014; Saunders et al., 2015; Abecassis et al., 2020), suggesting that the GPe may serve as an output nucleus within the cortico-basal ganglia loop." Lines 469-477 in the discussion "Rather than functioning as a closed loop or feedback circuit, the auditory cortico-basal ganglia loop may act as a gate by spontaneously suppressing higher auditory cortices. It is because PV-expressing GABAergic neurons in the GPe, which may exhibit high-frequency spontaneous firing (Ketzef and Silberberg, 2021), could serve as another gate by suppressing the non-lemniscal auditory pathway. Additionally, Lhx6-expressing GABAergic neurons in the GPe could directly suppress the TeA. Thus, when acoustic stimuli activate the auditory cortex, the spontaneous inhibition of GPe GABAergic neurons may be relieved through disinhibition via the auditory cortico-striato-pallidal pathway." Minor:

- The author should mention the brain atlas they followed.

We have added the following sentence in lines 168-169 "Histological analysis was conducted according to the mouse brain atlas to identify specific brain structures (Franklin and Paxinos, 2007)." - How the boundaries of MGB were determined by PKCδ immunoreactivity. I think the authors should add a supporting reference for the statement mentioned in lines 262-263.

We have added a supporting reference in lines 228-229 "Briefly, the boundaries of the MGB subdivisions were defined according to PKCδ immunoreactivity (Tomioka et al., 2023)." - The coordinates given to TeA did not show the lateral coordinates. Were these injections made directly from the surface of the brain's side? If yes, please provide the angle to the horizontal line the authors used to target the surface.

We have revised it as follows. "bregma, −3.0 mm; depth, 0.5 mm from the cortical surface at a 50-degree angle from the horizontal plane" - Line 328: Please add a supporting reference.

We have revised it as follows. "The CR immunoreactive neurons were mainly distributed in the MGd and PIN/PP (Fig. 4-1), which is consistent with previous reports (Lu et al., 2009)." - Figure 6: Which statistical tests have been used? How many animals are used? Also how many cells have been counted per animal and in total? Please mention the detailed tests.

We have described the statistical test as follows.

Lines 235-240 in the result "Six mice were used for quantitative measurement of Lhx6 immunoreactivity. Images were obtained using a confocal laser scanning microscope (FV3000) with a 40× objective lens at a 2048 × 2048 pixel resolution. We measured the intensity of Lhx6 immunoreactivity in the caudal GPe. We calculated the average intensities of Lhx6 immunoreactivity in PV-positive and PV-negative neurons in each animal. A two-tailed paired t-test was used for statistical analysis. Statistical significance was set at p<0.05. " Lines 347-349 in the result "Among Lhx6-immunoreactive neurons, PV-positive neurons exhibited weaker Lhx6 immunoreactivity than PV-negative neurons (Fig. 6G; p = 0.0131, two-tailed paired t-test, 37 {plus minus} 6 neurons across 6 animals)." -There is no labeling of the brain regions in the extended Figure 6.

We have added the labeling of brain regions in Fig. 12-1.

We trust that the revisions have addressed the reviewers' concerns and have strengthened the manuscript. Once again, we greatly appreciate the constructive feedback provided, and we look forward to your further evaluation of our revised manuscript.

Sincerely, Ryohei Tomioka (tomioka@kumamoto-u.ac.jp), Department of Sensory and Cognitive Physiology, Graduate School of Medical Sciences, Kumamoto University, Kumamoto 860-8556, Japan. Phone: +81-96-373-5060
