# Supplementary figures and images for: The External Globus Pallidus as the Hub of the Auditory Cortico-Basal Ganglia Loop
Source: eNeuro. 2024 Nov 26;11(11):ENEURO.0161-24.2024. doi: 10.1523/ENEURO.0161-24.2024 (PMC11594937; doi:10.1523/ENEURO.0161-24.2024)

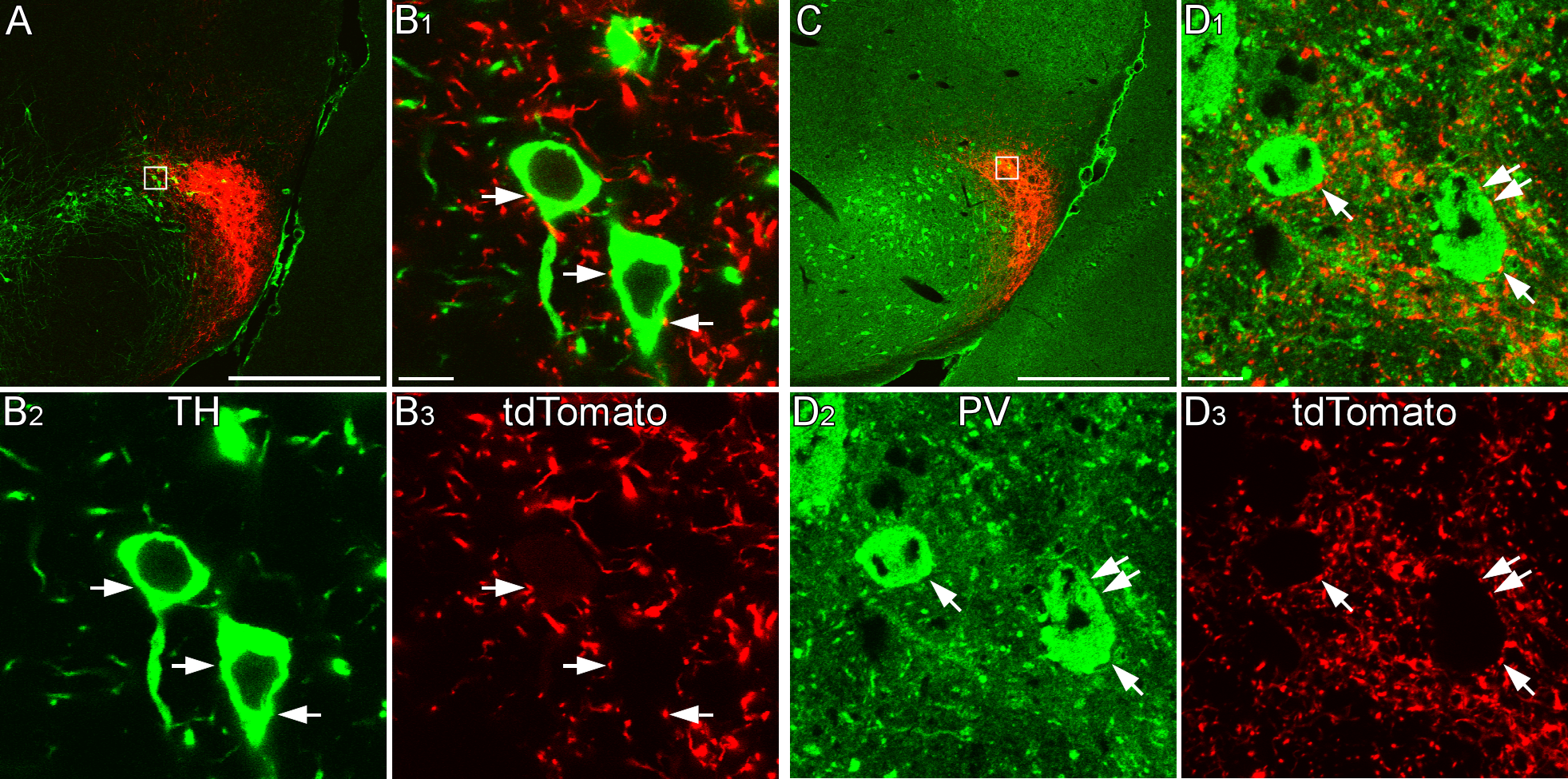

Supplement: Figure 1-1 — Axonal fibers of striatal neurons receiving corticostriatal projections from the primary auditory cortex. Striatal axonal fibers, which were labeled with tdTomato, formed close synaptic appositions (arrows) with TH-positive dopaminergic (A, B) or PV-positive GABAergic neurons (C, D) in the SNL. Scale bar = 500 µm in A and C, 10 µm in B and D. Download Figure 1-1, TIF file. [file eneuro-11-ENEURO.0161-24.2024-s001.tif]

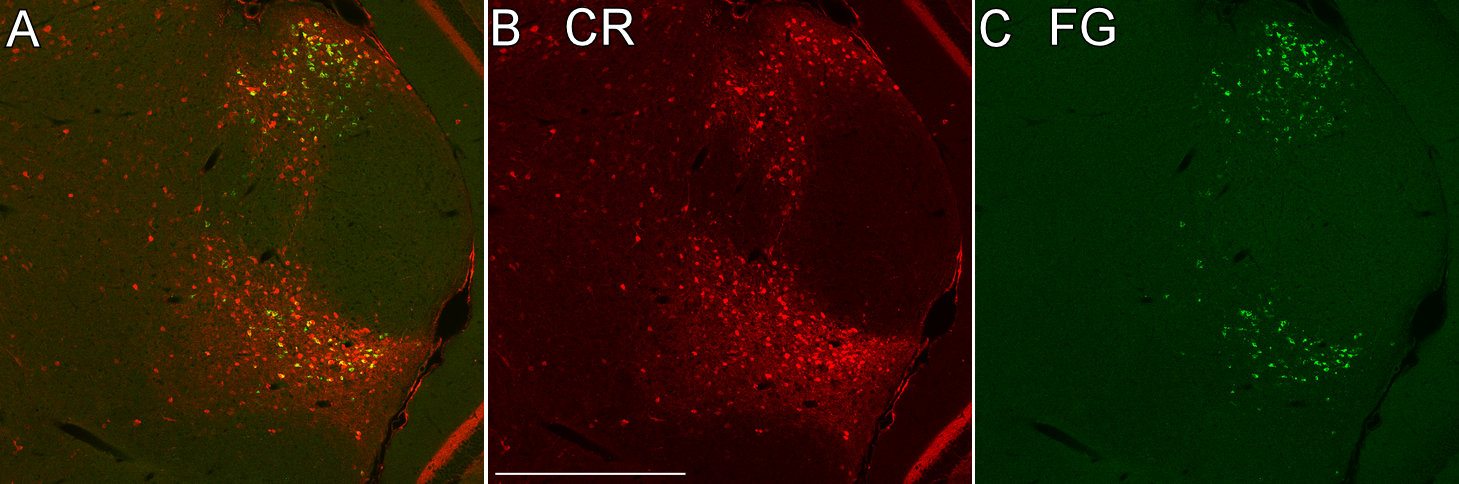

Supplement: Figure 4-1 — CR-immunoreactive neurons projecting to the temporal cortices. FG was injected into the temporal association area. Almost all FG-labeled neurons exhibited CR immunoreactivity. Scale bar = 500 µm. Download Figure 4-1, TIF file. [file eneuro-11-ENEURO.0161-24.2024-s002.tif]

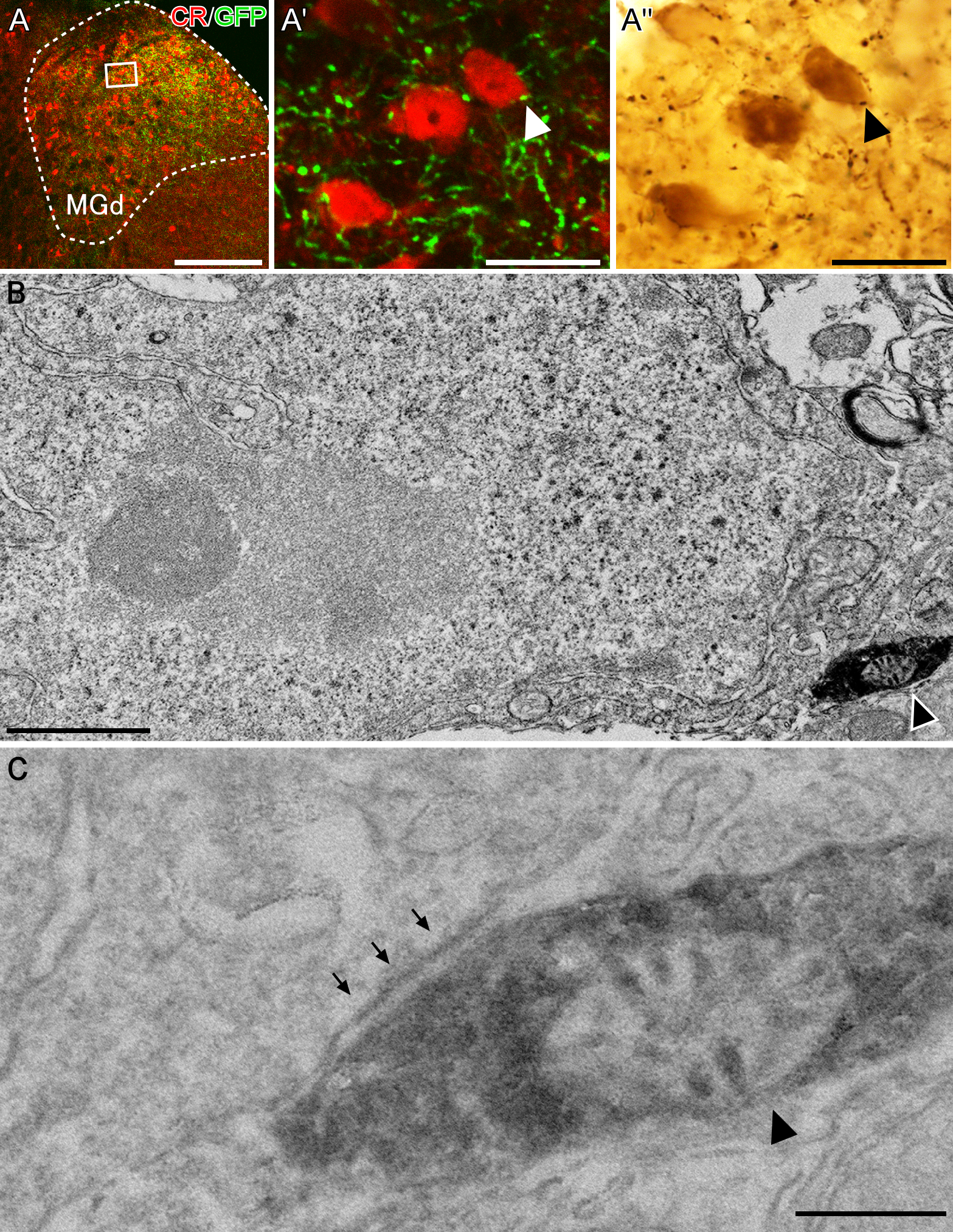

Supplement: Figure 4-2 — GPe GABAergic fibers forming synaptic contacts on CR-expressing neurons in the MGd. (A) CR-immunoreactive neurons in MGd and PIN/PP were surrounded by numerous GFP-expressing GPe GABAergic fibers. (A’) Higher magnification from the rectangle in (A). (A’’) GFP and CR immunoreactivities were developed by using DAB-nickel and DAB, respectively. (B) An EM image shows the axon terminal (arrowhead) corresponding to the arrowheads in (A’, A”). (C) The GFP-immunoreactive axon terminal formed a symmetrical synaptic contact on the CR-immunoreactive soma (arrows). Scale bars = 200 µm in A, 20 µm in A’ and A”, 1 µm in B, and 0.2 µm in C. Download Figure 4-2, TIF file. [file eneuro-11-ENEURO.0161-24.2024-s003.tif]

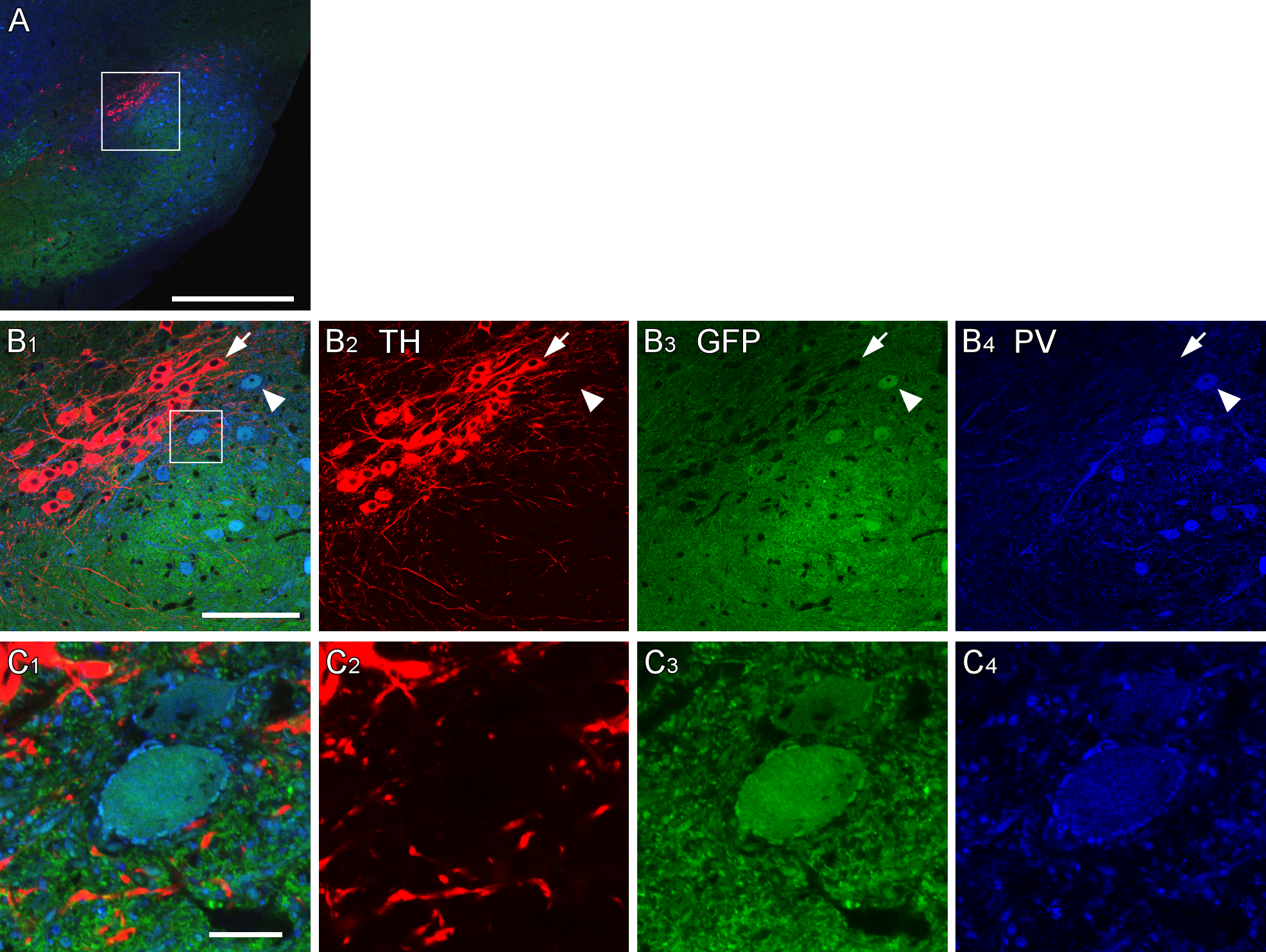

Supplement: Figure 5-1 — Neurochemical profiles in the substantia nigra. (A) GAD67-GFP mice were used in this experiment. Lower magnification of the substantia nigra. (B) Higher magnification from the rectangle in (A). TH-immunoreactive neurons were negative for GFP (arrow), whereas PV-immunoreactive neurons were positive for GFP (arrowhead). (C) Higher magnification from the rectangle in (B). We confirmed the dopaminergic and GABAergic neurons using TH and PV immunoreactivity, respectively. Scale bars = 500 µm in A, 100 µm in B, and 10 µm in C. Download Figure 5-1, TIF file. [file eneuro-11-ENEURO.0161-24.2024-s004.tif]

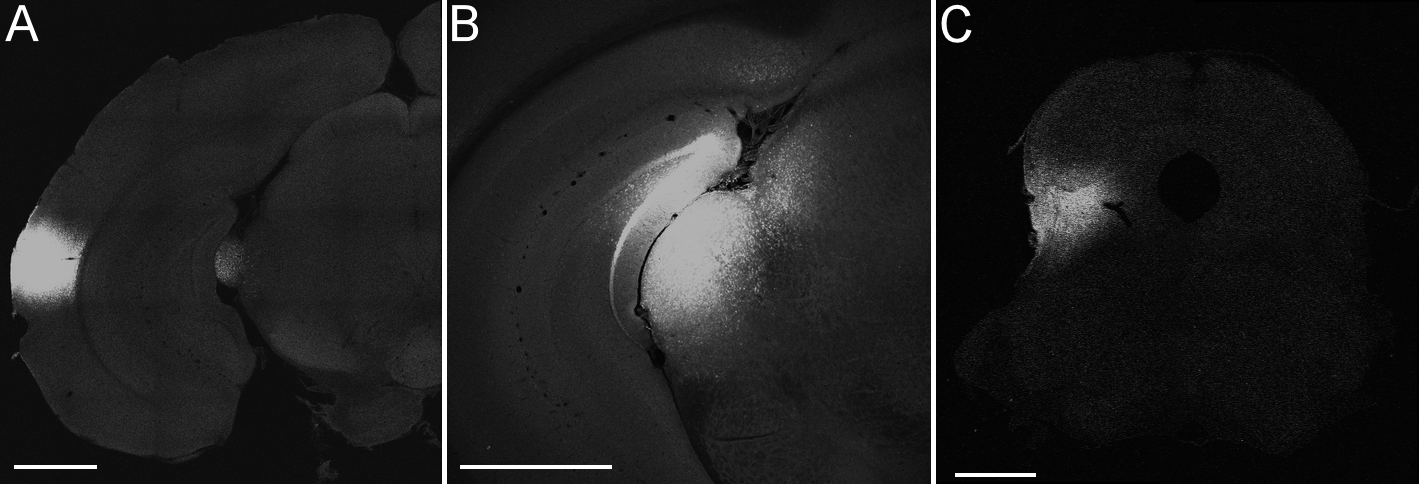

Supplement: Figure 11-1 — The representative FG injection sites. The main injection sites were TeA (A), MGB (B), and CnF (C). Scale bar = 1.0 mm. Download Figure 11-1, TIF file. [file eneuro-11-ENEURO.0161-24.2024-s005.tif]

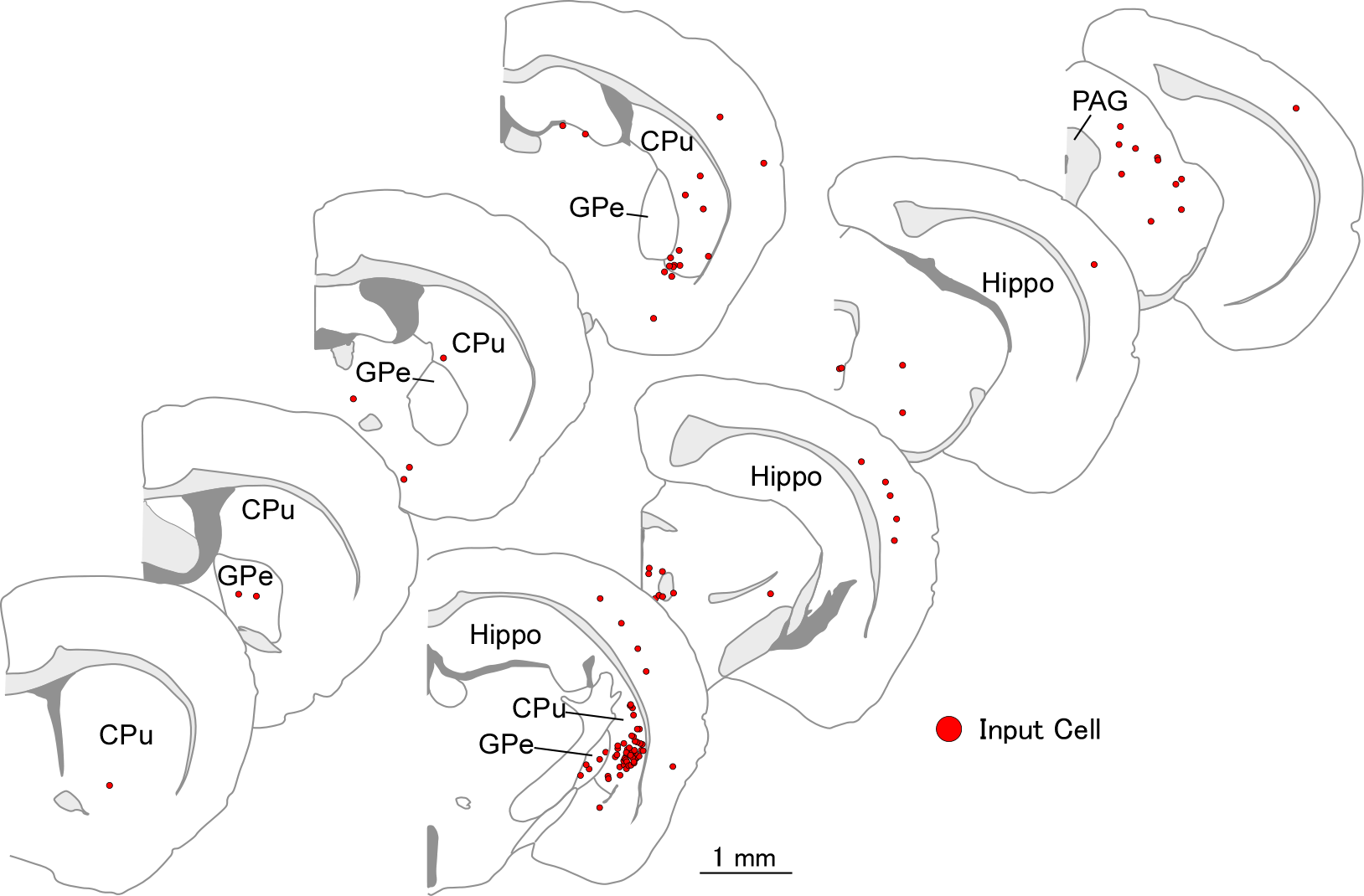

Supplement: Figure 12-1 — Distribution of presynaptic neurons of GPe GABAergic neurons. The parent neurons in the caudal GPe mainly received synaptic inputs from the TS. Hippocampus (Hippo), periaqueductal gray (PAG). Download Figure 12-1, TIF file. [file eneuro-11-ENEURO.0161-24.2024-s006.tif]

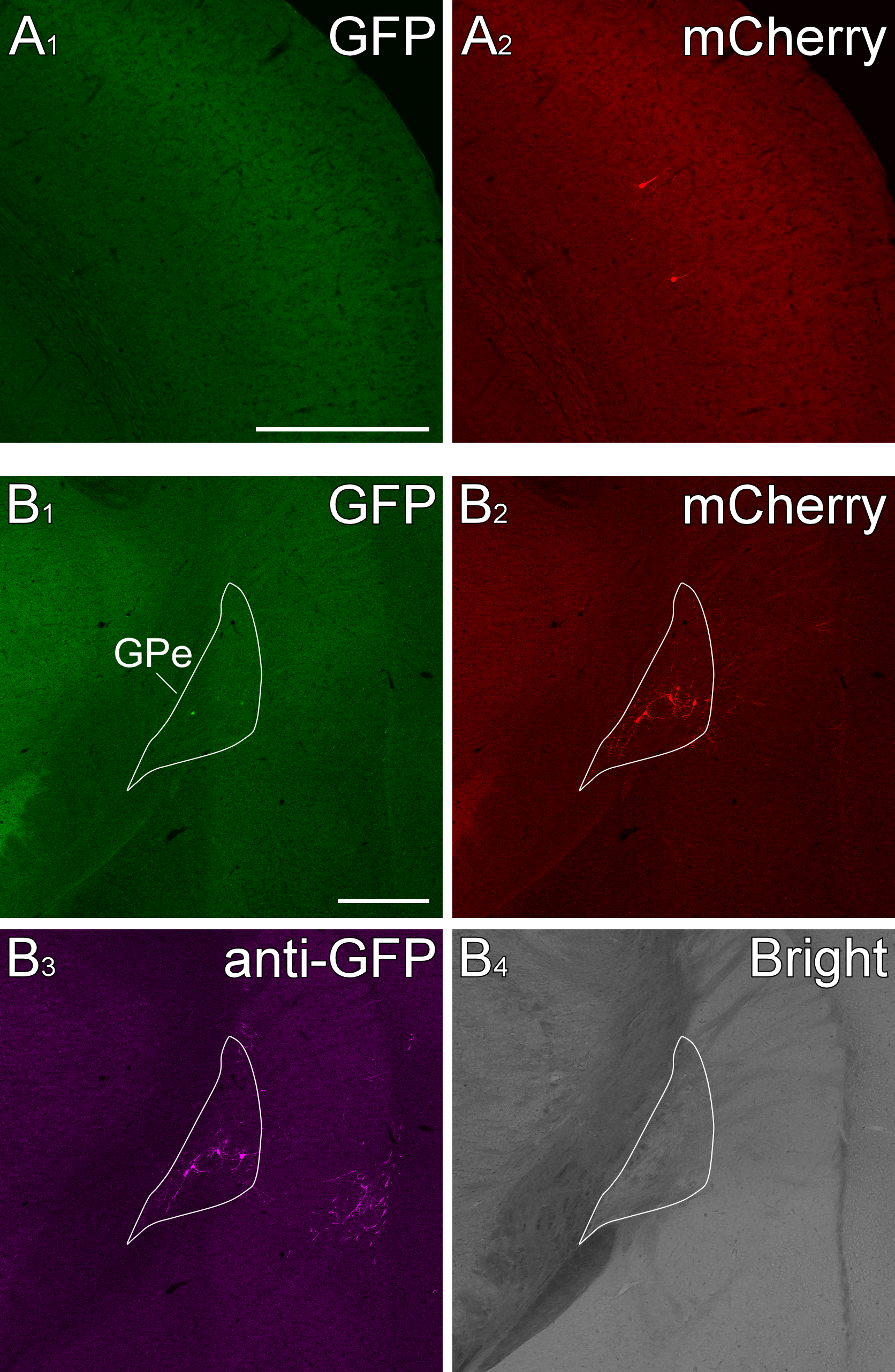

Supplement: Figure 12-2 — Two control experiments for pseudotyped rabies virus. （A）AAVretro-CA-FLEX-SADcvsG and AAVretro-EF1a-FLEX-GFP-2A-TVA were injected into the primary auditory cortex of wild mice, following the injection of pseudotyped rabies virus into the same area. Although no GFP-labeled neuron was found, a few mCherry-labeled neurons were around the injection site in the three animals. (B) AAVretro-EF1a-FLEX-GFP-2A-TVA were injected into the temporal association cortex of VGAT-Cre mice, following the injection of pseudotyped rabies virus into the caudal part of GPe. This is due to confirm the distribution of starter cells. Immunofluorescence of GFP allowed visualization of even weak GFP signals. Almost all of mCherry-labeled neurons were distributed in the caudal GPe. Scale bars = 500 µm. Download Figure 12-2, TIF file. [file eneuro-11-ENEURO.0161-24.2024-s007.tif]
